# Machine learning to design integral membrane channelrhodopsins for efficient eukaryotic expression and plasma membrane localization

**DOI:** 10.1371/journal.pcbi.1005786

**Published:** 2017-10-23

**Authors:** Claire N. Bedbrook, Kevin K. Yang, Austin J. Rice, Viviana Gradinaru, Frances H. Arnold

**Affiliations:** 1 Division of Biology and Biological Engineering; California Institute of Technology; Pasadena, California; United States of America; 2 Division of Chemistry and Chemical Engineering; California Institute of Technology; Pasadena, California; United States of America; The Pennsylvania State University, UNITED STATES

## Abstract

There is growing interest in studying and engineering integral membrane proteins (MPs) that play key roles in sensing and regulating cellular response to diverse external signals. A MP must be expressed, correctly inserted and folded in a lipid bilayer, and trafficked to the proper cellular location in order to function. The sequence and structural determinants of these processes are complex and highly constrained. Here we describe a predictive, machine-learning approach that captures this complexity to facilitate successful MP engineering and design. Machine learning on carefully-chosen training sequences made by structure-guided SCHEMA recombination has enabled us to accurately predict the rare sequences in a diverse library of channelrhodopsins (ChRs) that express and localize to the plasma membrane of mammalian cells. These light-gated channel proteins of microbial origin are of interest for neuroscience applications, where expression and localization to the plasma membrane is a prerequisite for function. We trained Gaussian process (GP) classification and regression models with expression and localization data from 218 ChR chimeras chosen from a 118,098-variant library designed by SCHEMA recombination of three parent ChRs. We use these GP models to identify ChRs that express and localize well and show that our models can elucidate sequence and structure elements important for these processes. We also used the predictive models to convert a naturally occurring ChR incapable of mammalian localization into one that localizes well.

## Author summary

A protein’s amino acid sequence determines how it will fold, traffic to subcellular locations, and carry out specific functions within the cell. Understanding this process would enable the design of protein sequences capable of useful functions; unfortunately, we cannot predict in detail how sequence encodes function. However, machine-learning models have the potential to infer the complex protein sequence-function relationship by identifying patterns or features that are important for function from sequences with known functions. We used machine learning to learn about and design membrane proteins (MPs). To function, a MP must be expressed, correctly folded in a lipid membrane, and trafficked to the proper cellular location. We built predictive, machine-learning models for this complex process from a set of >200 chimeric MPs and used them to design new sequences with optimal performance on the challenging task of membrane localization. This general approach to understanding and designing MPs could be broadly useful for important pharmaceutical and engineering MP targets.

## Introduction

As crucial components of regulatory and transport pathways, integral membrane proteins (MPs) are important pharmaceutical and engineering targets [1]. To be functional, MPs must be expressed and localized through a series of elaborate sub-cellular processes that include co-translational insertion, rigorous quality control, and multi-step trafficking to arrive at the correct topology in the correct sub-cellular location [2–4]. With such a complex mechanism for production, it is not surprising that MP engineering has been hampered by poor expression, stability, and localization in heterologous systems [5–7]. To overcome these limitations, protein engineers need a tool to predict how changes in sequence affect MP expression and localization. An accurate predictor would enable us to design and produce MP variants that express and localize correctly, a necessary first step in engineering MP function. A useful predictor would be sensitive to subtle changes in sequence that can lead to drastic changes in expression and localization. Our goal here was to develop data-driven models that predict the likelihood of a MP’s expression and plasma membrane localization using the amino acid sequence as the primary input.

For this study, we focus on channelrhodopsins (ChRs), light-gated ion channels that assume a seven transmembrane helix topology with a light-sensitive retinal chromophore bound in an internal pocket. This scaffold is conserved in both microbial rhodopsins (light-driven ion pumps, channels, and light sensors–type I rhodopsins) and animal rhodopsins (light-sensing G-protein coupled receptors–type II rhodopsins) [8]. Found in photosynthetic algae, ChRs function as light sensors in phototaxic and photophobic responses [9,10]. On photon absorption, ChRs undergo a multi-step photo-cycle that allows a flux of ions across the membrane and down the electrochemical gradient [11]. When ChRs are expressed transgenically in neurons, their light-dependent activity can stimulate action potentials, allowing cell-specific control over neuronal activity [12,13] and extensive applications in neuroscience [14]. The functional limitations of available ChRs have spurred efforts to engineer or discover novel ChRs [11]. The utility of a ChR, however, depends on its ability to express and localize to the plasma membrane in eukaryotic cells of interest, and changes to the amino acid sequence frequently abrogate localization [5]. A predictor for ChRs that express and localize would be of great value as a pre-screen for function.

The sequence and structural determinants for membrane localization have been a subject of much scientific investigation [15–17] and have provided some understanding of the MP sequence elements important for localization, such as signal peptide sequence, positive charge at the membrane–cytoplasm interface (the “positive-inside” rule [18]), and increased hydrophobicity in the transmembrane domains. However, these rules are of limited use to a protein engineer: there are too many amino acid sequences that follow these rules but still fail to localize to the plasma membrane (see Results). MP sequence changes that influence expression and localization are highly context-dependent: what eliminates localization in one sequence context has no effect in another, and subtle amino acid changes can have dramatic effects [5,16,19]. In short, sequence determinants of expression and localization are not captured by simple rules.

Accurate atomistic physics-based models relating a sequence to its level of expression and plasma membrane localization currently do not exist, in large measure due to the complexity of the process. Statistical models offer a powerful alternative. Statistical models are useful for predicting the outcomes of complex processes because they do not require prior knowledge of the specific biological mechanisms involved. That being said, statistical models can also be constructed to exploit prior knowledge, such as MP structural information. Statistical models can be trained using empirical data (in this case expression or localization values) collected from known sequences. During training, the model infers relationships between input (sequence) and output (expression or localization) that are then used to predict the properties of unmeasured sequence variants. The process of using empirical data to train and select statistical models is referred to as machine learning.

Machine learning has been applied to predicting various protein properties, including solubility [20,21], trafficking to the periplasm [22], crystallization propensity [23], and function [24]. Generally, these models are trained using large data sets composed of literature data from varied sources with little to no standardization of the experimental conditions, and trained using many protein classes (i.e. proteins with various folds and functions), because their aim is to identify sequence elements across all proteins that contribute to the property of interest. This generalist approach, however, is not useful for identifying subtle sequence features (i.e. amino acids or amino acid interactions) that condition expression and localization for a specific class of related sequences, the ChRs in this case. We focused our model building on ChRs, with training data collected from a range of ChR sequences under standardized conditions. We applied Gaussian process (GP) classification and regression [25] to build models that predict ChR expression and localization directly from these data.

In our previous work, GP models successfully predicted thermal stability, substrate binding affinity, and kinetics for several soluble enzymes [26]. Here, we asked whether GP modeling could accurately predict mammalian expression and localization for heterologous integral membrane ChRs and how much experimental data would be required. For a statistical model to make accurate predictions on a wide range of ChR sequences, it must be trained with a diverse set of ChR sequences [25]. We chose to generate a training set using chimeras produced by SCHEMA recombination, which was previously demonstrated to be useful for producing large sets (libraries) of diverse, functional chimeric sequences from homologous parent proteins [27]. We synthesized and measured expression and localization for only a small subset (0.18%) of sequences from the ChR recombination library. Here we use these data to train GP classification and regression models to predict the expression and localization properties of diverse, untested ChR sequences. We first made predictions on sequences within a large library of chimeric ChRs; we then expanded the predictions to sequences outside that set.

## Results

### The ChR training set

The design and characterization of the chimeric ChR sequences used to train our models have been published [5]; we will only briefly describe these results. Two separate, ten-block libraries were designed by recombining three parental ChRs (CsChrimsonR (CsChrimR) [28], C1C2 [29], and CheRiff [30]) with 45–55% amino acid sequence identity and a range of expression, localization, and functional properties (**S1 Fig**) [5]. Each chimeric ChR variant in these libraries is composed of blocks of sequence from the parental ChRs. These libraries were prepared by the SCHEMA algorithm to define sequence blocks for recombination that minimize the library-average disruption of tertiary protein structure [31,32]. One library swaps contiguous elements of primary structure (contiguous library), and the second swaps elements that are contiguous in the tertiary structure but not necessarily in the sequence (non-contiguous library [33]). The two libraries have similar, but not identical, element boundaries (**S1A Fig**) and were constructed in order to test whether one design approach was superior to the other (they gave similar results). These designs generate 118,098 possible chimeras (2 x 3^10^), which we will refer to as the recombination library throughout this paper. Each of these chimeras has a full N-terminal signal peptide from one of the three ChR parents.

Two hundred and eighteen chimeras from the recombination library were chosen as a training set, including all the chimeras with single-block swaps (chimeras consisting of 9 blocks of one parent and a single block from one of the other two parents) and multi-block-swap chimera sequences designed to maximize mutual information between the training set and the remainder of the chimeric library. Here, the ‘information’ a chimera has to offer is how its sequence, relative to all previously tested sequences, changes ChR expression and localization. By maximizing mutual information, we select chimera sequences that provide the most information about the whole library by reducing the uncertainty (Shannon entropy) of prediction for the remainder of the library, as described in [34,35]. The 112 single-block-swap chimeras in the training set have an average of 15 mutations from the most closely related parent, while the 103 multi-block-swap chimeras in the training set have an average of 73 mutations from the most closely related parent (**Table 1**). While the multi-block-swap chimeras provide the most sequence diversity to learn from, they are the least likely to express and localize given their high mutation levels. The single-block-swap chimeras offer less information to learn from due to their sequence redundancies with other chimeras in the training set, but are more likely to express and localize.

**Table 1:** Comparison of size, diversity, and localization properties of the training set and subsequent sets of chimeras chosen by models in the iterative steps of model development.

**Set**	**Count**	**Mutations** **mean ± stdev**	Percent with good localization*	**Localization** **mean ± stdev (x10**^**-3**^**)**
**training–parents**	3	0	100%	5.6 ± 3.0
**training–single-block swap**	112	15 ± 9	33%	3.2 ± 3.4
**training–multi-block swap**	103	73 ± 21	12%	1.5 ± 2.5
**Exploration**	16	69 ± 12	50%	4.8 ± 4.7
**verification–high performing**	4	29 ± 17	100%	8.0 ± 1.6
**verification–low performing**	7	67 ± 12	0%	0.89 ± 0.73
**optimization**	4	43 ± 6	100%	14 ± 3.5

* ‘good localization’ is localization at or above that of the lowest-performing parent, CheRiff

Genes for these sequences were synthesized and expressed in human embryonic kidney (HEK) cells, and their expression and membrane localization properties were measured (**S1B Fig**) [5]. The expression levels were monitored through a fluorescent protein (mKate) fused to the C-termini of the ChRs. Plasma-membrane localization was measured using the SpyTag/SpyCatcher labeling method, which exclusively labels ChR protein that has its N terminus exposed on the extracellular surface of the cell [36]. The training set sequences displayed a wide range of expression and localization properties. While the majority of the training set sequences express, only 33% of the single-block-swap chimeras localize well, and an even smaller fraction (12%) of the multi-block-swap chimeras localize well, emphasizing the importance of having a predictive model for membrane localization.

First we explored whether ChR chimera properties could be predicted based on basic biological properties, specifically, signal peptide sequence and hydrophobicity in the transmembrane (TM) domains. Each chimera in the library has one of the three parental signal peptides. Although the signal peptide sequence does affect expression and localization (**S2A Fig**), chimeras with any parental signal peptide can have high or low expression and localization. Thus, the identity of the signal peptide alone is insufficient for accurate predictions of the ChR chimera properties. We then calculated the level of hydrophobicity within the 7-TM domains of each chimera. With very weak correlation between increasing hydrophobicity and measured expression and localization (**S2B Fig**), hydrophobicity alone is also insufficient for accurate prediction of ChR chimera properties. These models do not accurately account for the observed levels of expression or localization (**S1 Fig**). Therefore, we need more expressive models to predict expression and localization from the amino acid sequences of these MPs.

### Using GP models to learn about ChRs

**Fig 1:**
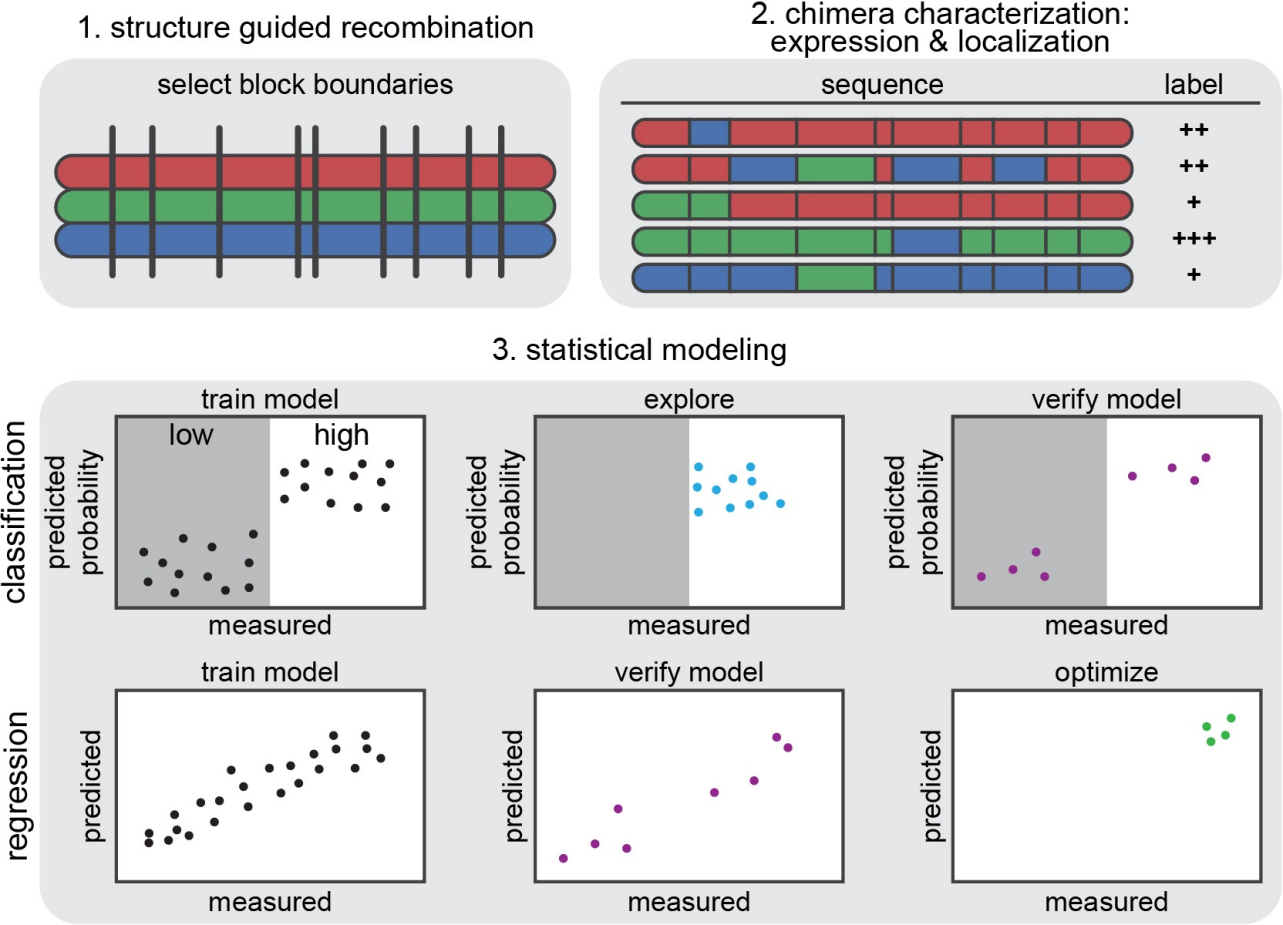
General approach to machine learning of protein (ChR) structure-function relationships: diversity generation, measurements on a training set, and modeling. (**1**) Structure-guided SCHEMA recombination is used to select block boundaries for shuffling protein sequences to generate a sequence-diverse ChR library starting from three parent ChRs (shown in red, green, and blue). (**2**) A subset of the library serves as the training set. Genes for these chimeras are synthesized and cloned into a mammalian expression vector, and the transfected cells are assayed for ChR expression and localization. (**3**) Two different models, classification and regression, are trained using the training data and then verified. The classification model is used to explore diverse sequences predicted to have ‘high’ localization. The regression model is used to design ChRs with optimal localization to the plasma membrane.

Our overall strategy for developing predictive machine-learning models is illustrated in **Fig 1**. The goal is to use a set of ChR sequences and their expression and localization measurements to train GP regression and classification models that describe how ChR properties depend on sequence and predict the behavior of untested ChRs. GP models infer predictive values from training examples by assuming that similar inputs (ChR sequence variants) will have similar outputs (expression or localization). We quantify the relatedness of inputs (ChR sequence variants) by comparing both sequence and structure. ChR variants with few differences are considered more similar than ChR variants with many differences. We define the sequence similarity between two chimeras by aligning them and counting the number of positions at which they are identical. For structural comparisons, a residue-residue ‘contact map’ was built for each ChR variant, where two residues are in contact if they have any non-hydrogen atoms within 4.5 Å. The maps were generated using a ChR parental sequence alignment and the C1C2 crystal structure, which is the only available ChR structure [29], with the assumption that ChR chimeras share the overall contact architecture observed in the C1C2 crystal structure. The structural similarity for any two ChRs was quantified by aligning the contact maps and counting the number of identical contacts [26]. Using these metrics, we calculated the sequence and structural similarity between all ChRs in the training set relative to one another (218 x 218 ChR comparisons).

These similarity functions are called kernel functions and specify how the functional properties of pairs of sequences are expected to covary (they are also known as covariance functions). In other words, the kernel is a measure of similarity between sequences, and we can draw conclusions about unobserved chimeras on the basis of their similarity to sampled points [25]. The model has high confidence in predicting the properties of sequences that are similar to previously sampled sequences, and the model is less confident in predicting the properties of sequences that are distant from previously sampled sequences.

To build a GP model, we must also specify how the relatedness between sequences will affect the property of interest, in other words how sensitive the ChR properties are to changes in relatedness as defined by the sequence/structure differences between ChRs. This is defined by the form of the kernel used. We tested three different forms of sequence and structure kernels: linear kernels, squared exponential kernels, and Matérn kernels (see Methods). These different forms represent the kinds of functions we expect to observe for the protein’s fitness landscape (i.e. the mapping of protein sequence to protein function). The linear kernel corresponds to a simple landscape where the effects of changes in sequence/structure are additive and there is no epistasis. The two non-linear kernels represent more rugged, complex landscapes where effects may be non-additive. Learning involves optimizing the form of the kernel and its hyperparameters (parameters that influence the form of kernel) to enable accurate predictions. The hyperparameters and the form of the kernel were optimized using the Bayesian method of maximizing the marginal likelihood of the resulting model. The marginal likelihood (i.e. how likely it is to observe the data given the model) rewards models that fit the training data well while penalizing model complexity to prevent overfitting.

Once trained with empirical data, the output of the GP regression model is a predicted mean and variance, or standard deviation, for any given ChR sequence variant. The standard deviation is an indication of how confident the model is in the prediction based on the relatedness of the new input relative to the tested sequences.

We used GP models to infer links between ChR properties and ChR sequence and structure from the training data. We first built GP binary classification models. In binary classification, the outputs are class labels i.e. ‘high’ or ‘low’ localization, and the goal is to use the training set data to predict the probability of a sequence falling into one of the two classes (**Fig 1**). We also built a GP regression model that makes real-valued predictions, i.e. amount of localized protein, based on the training data (**Fig 1**). After training these models, we verify that their predictions generalize to sequences outside of the training set. Once validated, these two models can be used in different ways. A classification model trained from localization data can be used to predict the probability of highly diverse sequences falling into the ‘high’ localization category (**Fig 1**). The classification model can only predict if a sequence has ‘high’ vs ‘low’ localization, and it cannot be used to optimize localization. The regression model, on the other hand, can be used to predict sequences with ‘optimal’ properties; for example, a regression model trained from localization data can predict untested sequences that will have very high levels of localization (**Fig 1**).

### Building GP classification models of ChR properties

**Fig 2:**
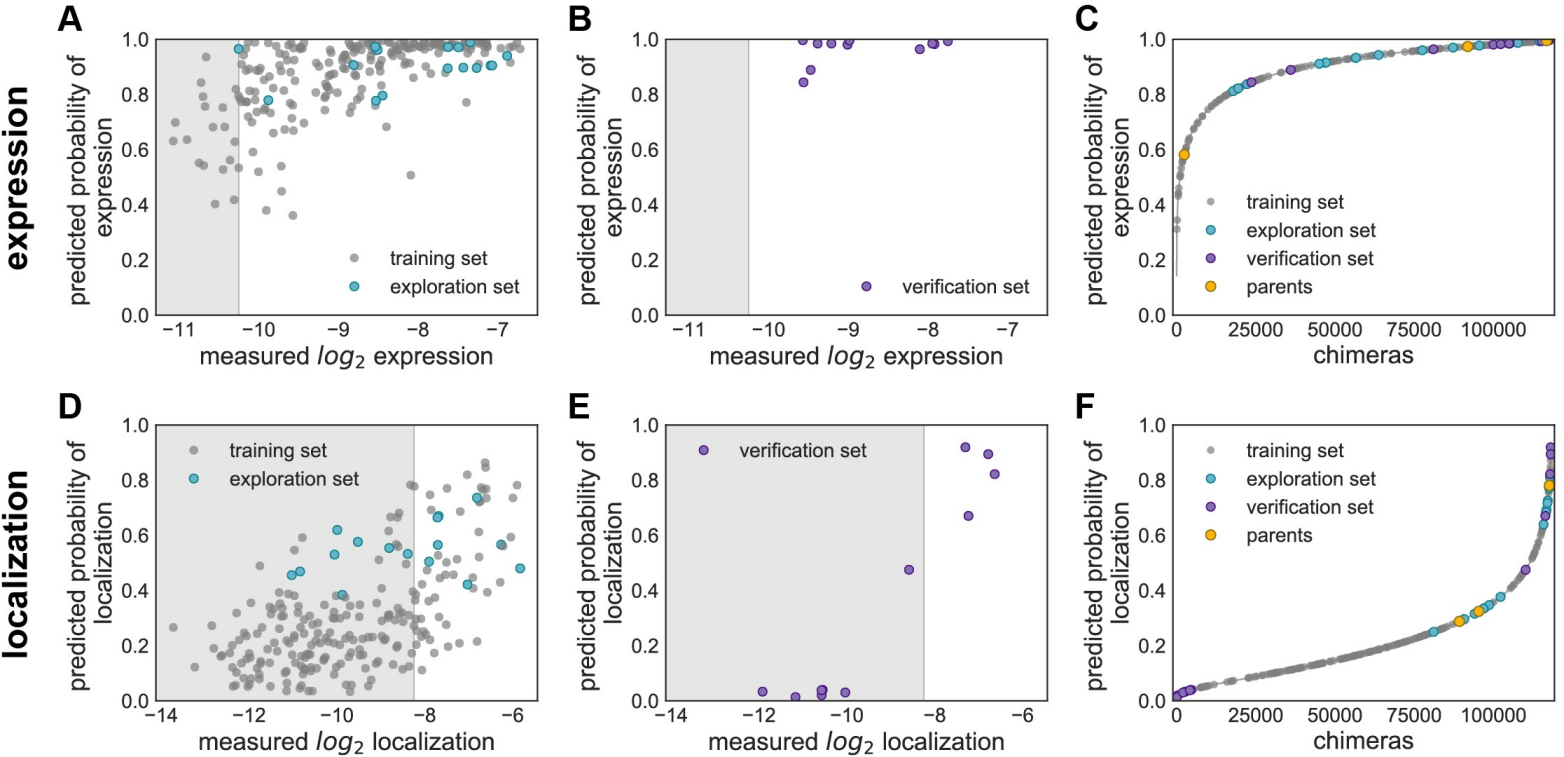
GP binary classification models for expression and localization. Plots of predicted probability vs measured properties are divided into ‘high’ performers (white background) and ‘low’ performers (gray background) for each property (expression and localization). (**A**) & (**D**) Predicted probability vs measured properties for the training set (gray points) and the exploration set (cyan points). Predictions for the training and exploration sets were made using LOO cross-validation. (**B**) & (**E**) Predicted probabilities vs measured properties for the verification set. Predictions for the verification set were made by a model trained on the training and exploration sets. (**C**) & (**F**) Predicted probability of ‘high’ expression, and localization for all chimeras in the recombination library (118,098 chimeras) made by models trained on the data from the training and exploration sets. The gray line shows all chimeras in the library, the gray points indicate the training set, the cyan points indicate the exploration set, the purple points indicate the verification set, and the yellow points indicate the parents. (**A**-**C**) Show expression and (**D**-**F**) show localization. For all plots, the measured property is plotted on a log_2_ scale.

The training set data (**S1 Fig**) were used to build a GP classification model that predicted which of the 118,098 chimeras in the recombination library would have ‘high’ vs ‘low’ expression, localization, and localization efficiency. The training set includes multi-block swaps chosen to be distant from other sequences in the training set in order to provide information on sequences throughout the recombination library. A sequence was considered ‘high’ if it performed at least as well as the lowest performing parent, and it was considered ‘low’ if it performed worse than the lowest performing parent. Because the lowest performing parent for expression and localization, CheRiff, is produced and localized in sufficient quantities for downstream functional studies, we believe this to be an appropriate threshold for ‘high’ vs ‘low’ performance. For all of the classification models (**Fig 2** and **S3 Fig**), we used kernels based on structural relatedness. For the expression classification model, we found that a linear kernel performed best, i.e. achieved the highest marginal likelihood. This suggests that expression is best approximated by an additive model weighting each of the structural contacts. Localization and localization efficiency required a non-linear kernel for the model to be predictive. This more expressive kernel allows for non-linear relationships and epistasis and also penalizes differing structural contacts more than the linear kernel. This reflects our intuitive understanding that localization is a more demanding property to tune than expression, with stricter requirements and a non-linear underlying fitness landscape.

**Fig 3:**
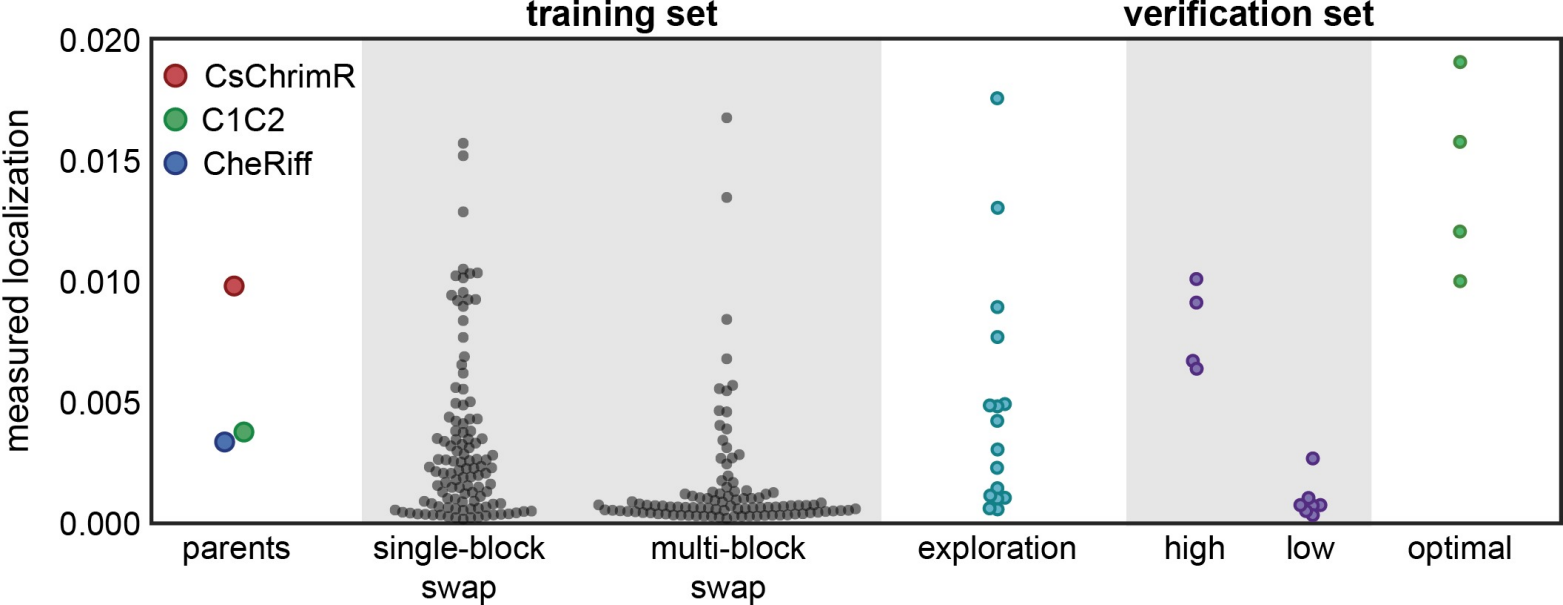
Comparison of measured membrane localization for each data set. Swarm plots of localization measurements for each data set compared with parents: training set, exploration set, verification set, and optimization set.

Most of the multi-block-swap sequences from the training set did not localize to the membrane [5]. We nonetheless want to be able to design highly mutated ChRs that localize well because these are most likely to have interesting functional properties. We therefore used the localization classification model to identify multi-block-swap chimeras from the library that had a high predicted probability (>0.4) of falling into the ‘high’ localizer category (**Fig 2D**). From the many multi-block-swap chimeras predicted to have ‘high’ localization, we selected a set of 16 highly diverse chimeras with an average of 69 amino acid mutations from the closest parent and called this the ‘exploration’ set (**S4 Fig**). We synthesized and tested these chimeras and found that the model had accurately predicted chimeras with good localization (**Fig 2** and **Fig 3**): 50% of the exploration set show ‘high’ localization compared to only 12% of the multi-block-swap sequences from the original training set, even though they have similar levels of mutation (**Table 1** and **S1 Data**) (chimeras in the exploration set have on average 69 ± 12 amino acid mutations from the closest parent, versus 73 ± 21 for the multi-block-swap chimeras in the training set). The classification model provides a four-fold enrichment in the number of chimeras that localize well when compared to randomly-selected chimeras with equivalent levels of mutation. This accuracy is impressive given that the exploration set was designed to be distant from any sequence the model had seen during training. The model’s performance on this exploration set indicates its ability to predict the properties of sequences distant from the training set.

The data from the exploration set were then used to better inform our models about highly diverse sequences that localize. To characterize the classification model’s performance, we calculated the area under the receiver operating characteristic (ROC) curve (AUC). A poorly performing model would not do better than random chance, resulting in an AUC of 0.5, while a model that perfectly separates the two classes will have an AUC of 1.0. The revised models achieved AUC up to 0.87 for “leave-one-out” (LOO) cross-validation, indicating that there is a high probability that the classifiers will accurately separate ‘high’ and ‘low’ performing sequences for the properties measured. The AUC is 0.83 for localization, 0.77 for localization efficiency and 0.87 for expression for LOO cross-validation predictions (**S5 Fig**).

To further test the models, we then built a verification set of eleven chimeras, designed using the localization model. This verification set was composed of four chimeras predicted to be highly likely to localize, six chimeras predicted to be very unlikely to localize, and one chimera with a moderate predicted probability of localizing (**S4 Fig**). The measured localization (**Fig 2E**) and localization efficiency (**S3B Fig**) of the chimeras in the verification set show clear differences, ‘high’ vs ‘low’, consistent with the model predictions (**Table 1** and **S1 Data**). The verification sets consist exclusively of chimeras with ‘high’ measured expression, which is consistent with the model’s predictions (**Fig 2B**). The model perfectly classifies the eleven chimeras as either ‘high’ or ‘low’ for each property (expression, localization, or localization efficiency) as shown in plots of predicted vs measured properties (**Fig 2B** and **2E** and **S3B Fig**) and by perfect separation in ROC curves i.e. AUC = 1.0 (**S5 Fig**). These models are powerful tools that can confidently predict whether a chimera will have 'high' or 'low' expression (**Fig 2C**), localization (**Fig 2F**), and localization efficiency (**S3C Fig**). Of the 118,098 chimeras in the recombination library, 6,631 (5.6%) are predicted to have a probability > 0.5 of 'high' localization, whereas the vast majority of chimeras (99%) are predicted to have a probability > 0.5 of 'high' expression.

### Building a regression model for ChR localization

**Fig 4:**
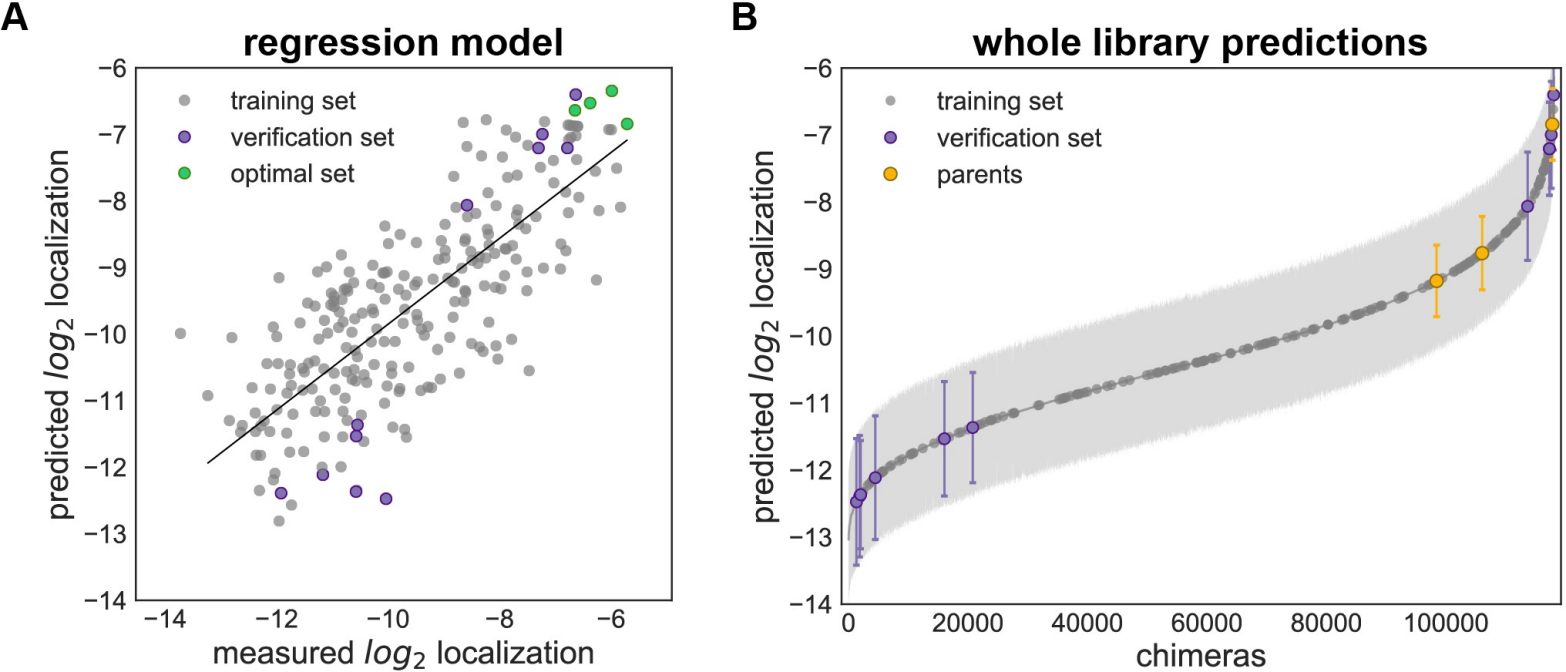
GP regression model for localization. (**A**) Predicted vs measured localization for the combined training and exploration sets (gray points), verification set (purple points), and the optimal set (green points). Predictions for the training and exploration sets were made using LOO cross-validation; predictions for the verification and optimal sets were made by a model trained on data from the training and exploration sets. There is clear correlation between predicted and measured localization. The combined training and exploration sets showed good correlation (R > 0.73) as did the verification set (R > 0.9). (**B**) Predicted localization values of all chimeras in the recombination library (118,098 chimeras) based on the GP regression model trained on the training and exploration sets. The gray line shows all chimeras in the library, the gray points indicate the training set and exploration sets, the purple points indicate the verification set, and the yellow points indicate the parents. Error bars (light gray shading) show the standard deviation of the predictions. For all plots, the predicted and measured localization are plotted on a log_2_ scale.

The classification model predicts the probability that a sequence falls into the ‘high’ localizer category, but does not give a quantitative prediction as to how well it localizes. Our next goal was to design chimera sequences with optimal localization. Localization is considered optimal if it is at or above the level of CsChrimR, the best localizing parent, which is more than adequate for *in vivo* applications using ChR functionality to control neuronal activity [28]. A regression model for ChR plasma membrane localization is required to predict sequences that have optimal levels of localization. We used the localization data from the training and exploration sets to train a GP regression model (**Fig 4A**). The diversity of sequences in the training data allows the model to generalize well to the remainder of the recombination library. For this regression model, we do not use all of the features from the combined sequence and structure information; instead, we used L1 linear regression to select a subset of these features. The L1 linear regression identifies the sequence and structural features that most strongly influence ChR localization. Using this subset of features instead of all of the features improved the quality of the predictions (as determined by cross-validation). This indicates that not all of the residues and residue-residue contacts have a large influence on localization of ChR. We then used a kernel based on these chosen features (specific contacts and residues) for GP regression. The regression model for localization showed strong predictive ability as indicated by the strong correlation between predicted and measured localization for LOO cross-validation (correlation coefficient, R > 0.76) (**Fig 4A**). This was further verified by the strong correlation between predicted and measured values for the previously-discussed verification set (R > 0.9) (**Fig 4A**). These cross-validation results suggest that the regression model can be used to predict chimeras with optimal localization.

We used the localization regression model to predict ChR chimeras with optimal localization using the Lower Confidence Bound (LCB) algorithm, in which the predicted mean minus the predicted standard deviation (LB1) is maximized [37]. The LCB algorithm maximally exploits the information learned from the training set by finding sequences the model is most certain will be good localizers. The regression model was used to predict the localization level and standard deviation for all chimeras in the library, and from this the LB1 was calculated for all chimeras (**Fig 4B**). We selected four chimeras whose LB1 predictions for localization were ranked in the top 0.1% of the library (**S4 Fig**). These were constructed and tested (**Fig 3** and **S6 Fig** and **S1 Data**). Measurements showed that they all localize as well as or better than CsChrimR (**Fig 3** and **Fig 4A** and **Table 1**). Cell population distributions of the optimal set show properties similar to the CsChrimR parent, with one chimera showing a clear shift in the peak of the distribution towards higher levels of localization (**S7 Fig**). These four sequences differ from CsChrimR at 30 to 50 amino acids (**S4 Fig**).

We were interested in how predictive the GP localization models could be with fewer training examples. To assess the predictive ability of the GP models as a function of training set size, we sampled random sets of training sequences from the dataset, trained models on these random sets, then evaluated the model’s performance on a selected test set (**S8 Fig**). As few as 100 training examples are sufficient for accurate predictions for both the localization regression and classification models. This analysis shows that the models would have been predictive with even fewer training examples than we chose to use.

### Sequence and structure features that facilitate prediction of ChR expression and localization

In developing the GP regression model for localization, we used L1-regularized linear regression to identify a limited set of sequence and structural features that strongly influence ChR localization (**Fig 4**). These features include both inter-residue contacts and individual residues and offer insight into the structural determinants of ChR localization. To better gauge the relative importance of these features, L2-regularized linear regression was used to calculate the positive and negative feature weights, which are proportional to each feature’s inferred contribution to localization. While not as predictive as the GP regression model because it cannot account for higher-order interactions between features, this linear model has the advantage of being interpretable.

**Fig 5:**
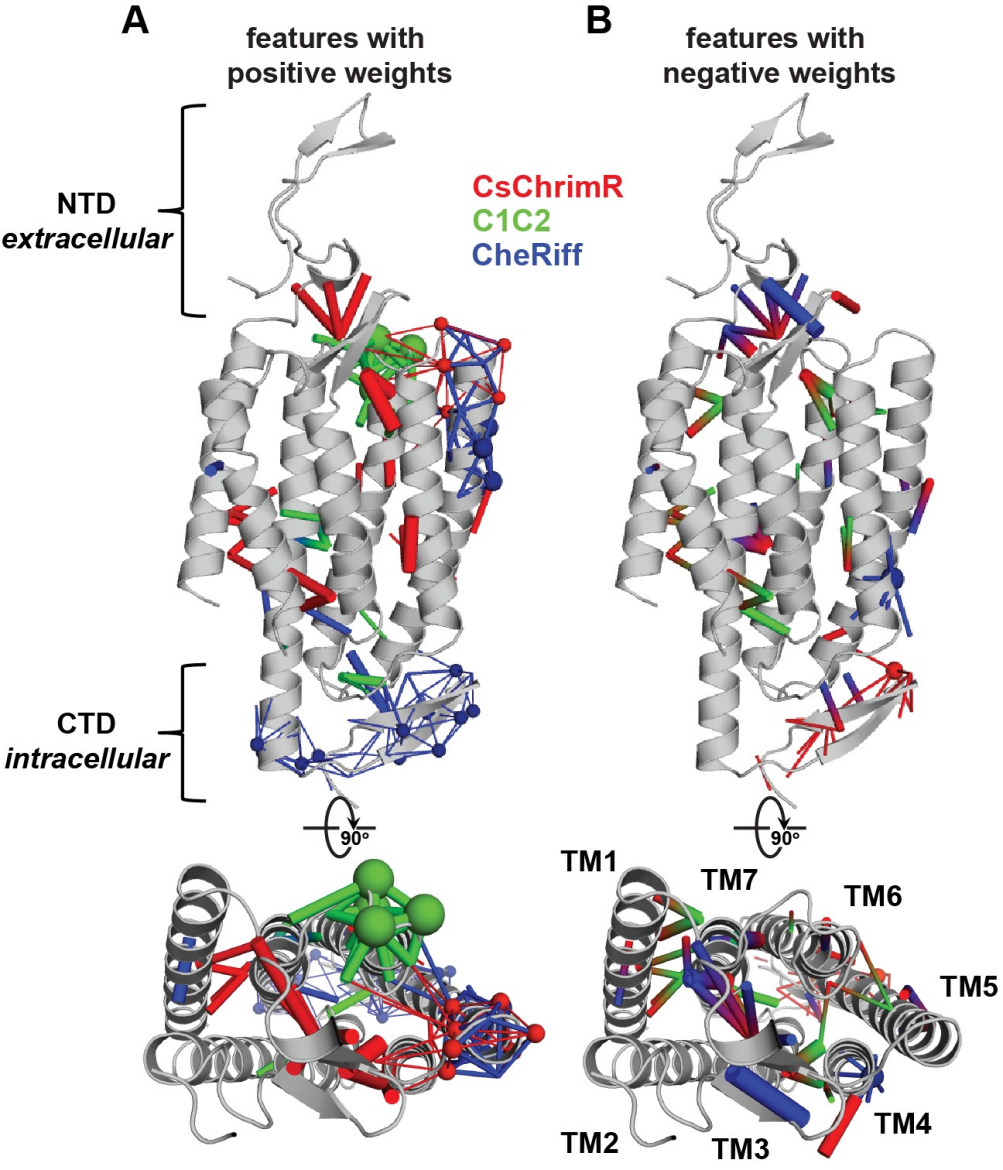
Sequence and structural contact features important for prediction of ChR localization. Features with positive (**A**) and negative (**B**) weights are displayed on the C1C2 crystal structure (grey). Features can be residues (spheres) or contacts (sticks) from one or more parent ChRs. Features from CsChrimR are shown in red, features from C1C2 are shown in green, and features from CheRiff are shown in blue. In cases where a feature is present in two parents, the following color priorities were used for consistency: red above green above blue. Sticks connect the beta carbons of contacting residues (or alpha carbon in the case of glycine). The size of the spheres and the thickness of the sticks are proportional to the parameter weights. Two residues in contact can be from the same or different parents. Single-color contacts occur when both contributing residues are from the same parent. Multi-color contacts occur when residues from different parents are in contact. The N-terminal domain (NTD), C-terminal domain (CTD), and the seven transmembrane helices (TM1-7) are labeled.

When mapped onto the C1C2 structure, these features highlight parts of the ChR sequence and structural contacts that are important for ChR localization to the plasma membrane (**Fig 5**). Both beneficial and deleterious features are distributed throughout the protein, with no single feature dictating localization properties (**Fig 5**). Clusters of heavily weighted positive contacts suggest that having structurally proximal CsChrimR-residue pairs are important in the N-terminal domain (NTD), between the NTD and TM4, between TM1 and TM7, and between TM3 and TM7. CsChrimR residues at the extracellular side of TM5 also appear to aid localization, although they are weighted less than CheRiff residues in the same area. Beneficial CheRiff contacts and residues are found in the C-terminal domain (CTD), the interface between the CTD and TM5-6, and in TM1. C1C2 residues at the extracellular side of TM6 are also positively weighted for localization, as are C1C2 contacts between the CTD and TM3-4 loop. From the negatively weighted contacts, it is clear that total localization is harmed when CheRiff contributes to the NTD or the intracellular half of TM4 and when CsChrimR contributes to the CTD. Interestingly, positive contacts were formed between TM6 from C1C2 and TM7 from CheRiff, but when the contributions were reversed (TM6 from CheRiff TM7 from C1C2) or if CsChrimR contributed TM6, strong negative weights were observed. Not surprisingly, the sequence and structure of optimal localizers predicted by GP regression (**Fig 4**) largely agree with the L2 weights (**S9 Fig**).

Using this strategy for model interpretation (L1 regression for feature selection followed by L2 regression), we can also weight the contributions of residues and contacts for ChR expression (**S10 Fig** and **S11 Fig**). There is some overlap between the heavily weighted features for ChR expression and the features for localization, which is expected because more protein expressed means more protein available for localization. For example, both expression and localization models seem to prefer the NTD from CsChrimR and the extracellular half of TM6 from C1C2, and both disfavor the NTD and the intra-cellular half of TM4 from CheRiff. While the heavily-weighted expression features are limited to these isolated sequence regions, localization features are distributed throughout the protein. Moreover, the majority of heavily-weighted features identified for expression are residues rather than contacts. This is in contrast to those weighted features identified for localization, which include heavily-weighted residues and structural contacts. This suggests that sequence is more important in determining expression properties, which is consistent with the largely sequence-dependent mechanisms associated with successful translation and insertion into the ER membrane. In contrast, both sequence and specific structural contacts contribute significantly to whether a ChR will localize to the plasma membrane. Our results demonstrate that the model can ‘learn’ the features that contribute to localization from the data and make accurate predictions on that property.

### Using the GP regression model to engineer novel sequences that localize

**Fig 6:**
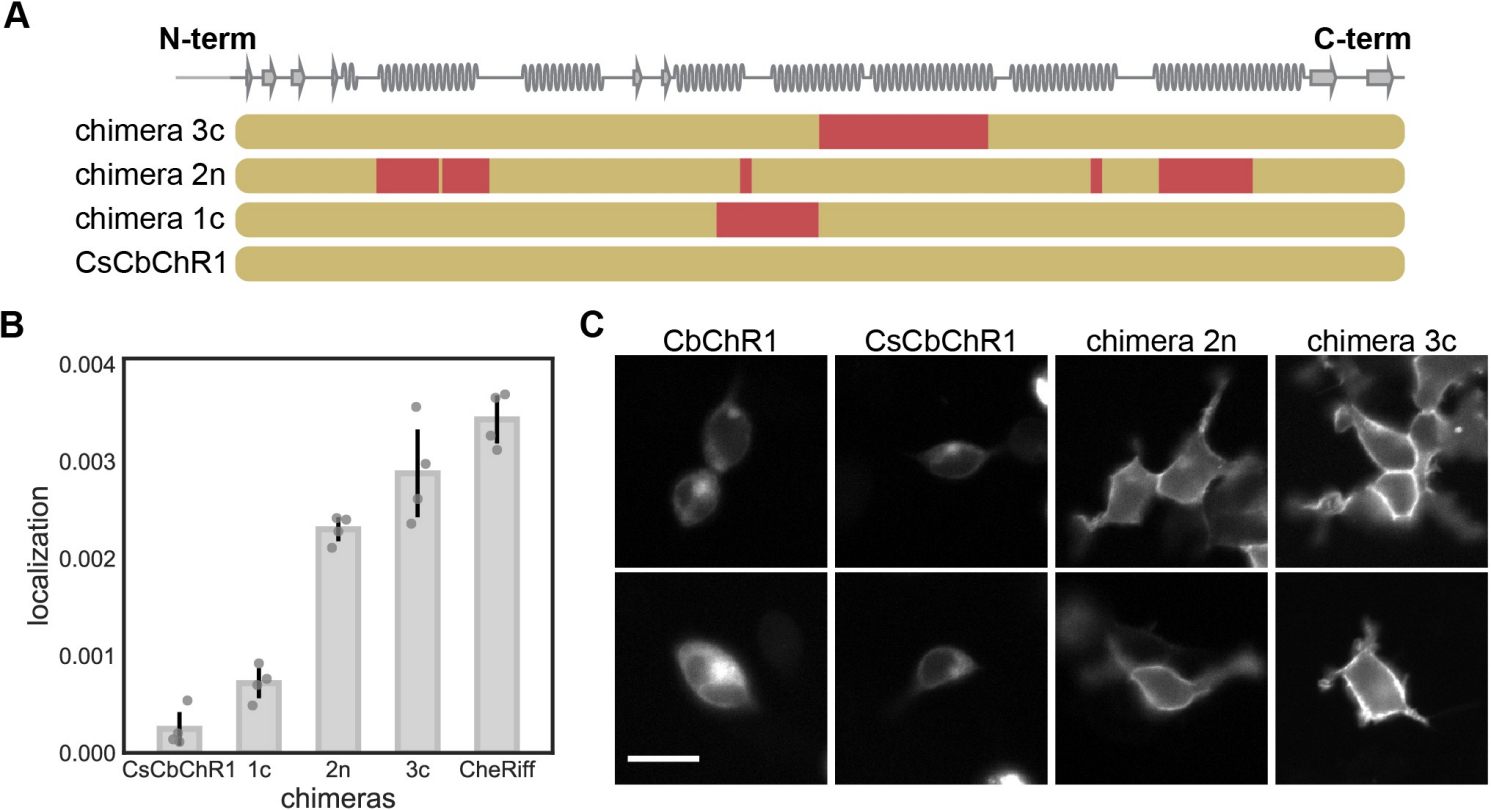
GP regression model enables engineering of localization in CbChR1. (**A**) Block identities of the CsCbChR1 chimeras. Each row represents a chimera. Yellow represents the CbChR1 parent and red represents the CsChrimR parent. Chimeras 1c, 2n, and 3c have 4, 21, and 17 mutations with respect to CsCbChR1, respectively. (**B**) Plot of measured localization of CsCbChR1 compared to three CsCbChR1 single-block-swap chimeras and the CheRiff parent. (**C**) Two representative cell images of mKate expression of CbChR1 and CsCbChR1 compared with top-performing CsCbChR1 single-block-swap chimeras show differences in ChR localization properties–chimera 2n and chimera 3c clearly localize to the plasma membrane. Scale bar: 20 μm.

We next tested the ChR localization regression model for its ability to predict plasma-membrane localization for ChR sequences outside the recombination library. For this, we chose a natural ChR variant, CbChR1, that expresses in HEK cells and neurons but does not localize to the plasma membrane and thus is non-functional [28]. CbChR1 is distant from the three parental sequences, with 60% identity to CsChrimR and 40% identity to CheRiff and C1C2. We optimized CbChR1 by introducing minor amino acid changes predicted by the localization regression model to be beneficial for membrane localization. To enable measurement of CbChR1 localization with the SpyTag-based labeling method, we substituted the N-terminus of CbChR1 with the CsChrimR N-terminus containing the SpyTag sequence downstream of the signal peptide to make the chimera CsCbChR1 [36]. This block swap did not change the membrane localization properties of CbChR1 (**Fig 6C**). Using the regression model, we predicted localization levels for all the possible single-block swaps from the three library parents (CsChrimR, C1C2 and CheRiff) into CsCbChR1 and selected the four chimeras with the highest Upper Confidence Bound (UCB). These chimeras have between 4 and 21 mutations when compared with CsCbChR1. Unlike the LCB algorithm, which seeks to find the safest optimal choices, the UCB algorithm balances exploration and exploitation by maximizing the sum of the predicted mean and standard deviation.

The selected chimeras were assayed for expression, localization, and localization efficiency (**S1 Data**). One of the four sequences did not express; the other three chimeras expressed and had higher localization levels than CsCbChR1 (**Fig 6B**). Two of the three had localization properties similar to the CheRiff parent (**Fig 6B**). Images of the two best localizing chimeras illustrate the enhancement in localization when compared with CbChR1 and CsCbChR1 (**Fig 6C** and **S12 Fig**). This improvement in localization was achieved through single-block swaps from CsChrimR (17 and 21 amino acid mutations) (**Fig 6A**). These results suggest that this regression model can accurately predict minor sequence changes that will improve the membrane localization of natural ChRs.

## Discussion

The ability to differentiate the functional properties of closely related sequences is extremely powerful for protein design and engineering. This is of particular interest for protein types that have proven to be more recalcitrant to traditional protein design methods, e.g. MPs. We show here that integral membrane protein expression and plasma membrane localization can be predicted for novel, homologous sequences using moderate-throughput data collection and advanced statistical modeling. We have used the models in four ways: 1) to accurately predict which diverse, chimeric ChRs are likely to express and localize at least as well as a moderately-performing native ChR; 2) to design ChR chimeras with optimized membrane localization that matched or exceeded the performance of a very well-localizing ChR (CsChrimR); 3) to identify the structural interactions (contacts) and sequence elements most important for predicting ChR localization; and 4) to identify limited sequence changes that transform a native ChR from a non-localizer to a localizer.

Whereas 99% of the chimeras in the recombination library are predicted to express in HEK cells, only 5.6% are predicted to localize to the membrane at levels equal to or above the lowest parent (CheRiff). This result shows that expression is robust to recombination-based sequence alterations, whereas correct plasma-membrane localization is much more sensitive. The model enables accurate selection of the rare, localization-capable, proteins from the nearly 120,000 possible chimeric library variants. In future work we will show that this diverse set of several thousand variants predicted to localize serves as a highly enriched source of functional ChRs with novel properties.

Although statistical models generalize poorly as one attempts to make predictions on sequences distant from the sequences used in model training, we show that it is possible to train a model that accurately distinguishes between closely related proteins. The tradeoff between making accurate predictions on subtle sequence changes vs generalized predictions for significantly different sequences is one we made intentionally in order to achieve accurate predictions for an important and interesting class of proteins. Accurate statistical models, like the ones described in this paper, could aid in building more expressive physics-based models.

This work details the steps in building machine-learning models and highlights their power in predicting desirable protein properties that arise from the intersection of multiple cellular processes. Combining recombination-based library design with statistical modeling methods, we have scanned a highly functional portion of protein sequence space by training on only 218 sequences. Model development through iterative training, exploration, and verification has yielded a tool that not only predicts optimally performing chimeric proteins, but can also be applied to improve related ChR proteins outside the library. As large-scale gene synthesis and DNA sequencing become more affordable, machine-learning methods such as those described here will become ever more powerful tools for protein engineering offering an alternative to high-throughput assay systems.

## Materials and methods

The design, construction, and characterization of recombination library chimeras is described in Bedbrook *et al*. [5]. Briefly, HEK 293T cells were transfected with purified ChR variant DNA using Fugene6 reagent according to the manufacturer’s recommendations. Cells were given 48 hours to express before expression and localization were measured. To assay localization level, transfected cells were subjected to the SpyCatcher-GFP labeling assay, as described in Bedbrook *et al*. [36]. Transfected HEK cells were then imaged for mKate and GFP fluorescence using a Leica DMI 6000 microscope (for cell populations) or a Zeiss LSM 780 confocal microscope (for single cells: **S12 Fig**). Images were processed using custom image processing scripts for expression (mean mKate fluorescence intensity) and localization (mean GFP fluorescence intensity). All chimeras were assayed under identical conditions.

For each chimera, net hydrophobicity was calculated by summing the hydrophobicity of all residues in the TM domains. The C1C2 crystal structure was used to identify residues within TM domains (**S2B Fig**), and the Kyte & Doolittle amino acid hydropathicity scale [38] was used to score residue hydrophobicity.

### GP modeling

Both the GP regression and classification modeling methods applied in this paper are based on work detailed in [26]. Romero *et al*. applied GP models to predict protein functions and also defined protein distance using a contact map. We have expanded on this previous work. Regression and classification were performed using open-source packages in the SciPy ecosystem [39–41]. Below are specifics of the GP regression and classification methods used in this paper. The hyperparameters and the form of the kernel were optimized using the Bayesian method of maximizing the marginal likelihood of the resulting model.

#### GP regression

In regression, the problem is to infer the value of an unknown function *f*(*x*) at a novel point x_*_ given observations *y* at inputs *X*. Assuming that the observations are subject to independent identically distributed Gaussian noise with variance $\sigma_{n}^{2}$, the posterior distribution of f_*_ = *f*(x_*_) for Gaussian process regression is Gaussian with mean

$${\bar{f}}_{*}=k_{*}^{T}{\left(K+\sigma_{n}^{2}I\right)}^{-1}y$$ (1)

and variance

$$v_{*}=k(x_{*},x_{*})-k_{*}^{T}{\left(K+\sigma_{n}^{2}I\right)}^{-1}k_{*}$$ (2)

Where

1. *K* is the symmetric, square covariance matrix for the training set, where *K*_*ij*_ = *k*(*x*_*i*_,*x*_*j*_) for *x*_*i*_ and *x*_*j*_ in the training set.
2. k_*_ is the vector of covariances between the novel input and each input in the training set, where k_*i_ = *k*(x_*_,*x*_*i*_).

We found that results could be improved by first performing feature selection with L1-regularized linear regression and then only training the GP model on features with non-zero weights in the L1 regression. The hyperparameters in the kernel functions, the noise hyperparameter *σ*_*p*_ and the regularization hyperparameter were determined by maximizing the log marginal likelihood:

$$\log\;p(y|X)=-\frac{1}{2}y^{T}{\left(K+\sigma_{n}^{2}I\right)}^{-1}y-\frac{1}{2}\log\left|K+\sigma_{n}^{2}I\right|-\frac{n}{2}\log2\pi$$ (3)

where *n* is the dimensionality of the inputs.

#### GP classification

In binary classification, instead of continuous outputs *y*, the outputs are class labels *y*_*i*_ ∈ {+1,−1}, and the goal is to use the training data to make probabilistic predictions *π*(x_*_) = *p*(y_*_ = +1|x_*_). Unfortunately, the posterior distribution for classification is analytically intractable. We use Laplace's method to approximate the posterior distribution. There is no noise hyperparameter in the classification case. Hyperparameters in the kernels are also found by maximizing the marginal likelihood.

#### GP kernels for modeling proteins

Gaussian process regression and classification models require kernel functions that measure the similarity between protein sequences. A protein sequence *s* of length *l* is defined by the amino acid present at each location. This information can be encoded as a binary feature vector *x*_*se*_ that indicates the presence or absence of each amino acid at each position. The protein's structure can be represented as a residue-residue contact map. The contact-map can be encoded as a binary feature vector *x*_*st*_ that indicates the presence or absence of each possible contacting pair. The sequence and structure feature vectors can also be concatenated to form a sequence-structure feature vector.

We considered three types of kernel functions *k*(*s*_*i*_,*s*_*j*_): linear kernels, squared exponential kernels, and Matérn kernels. The linear kernel is defined as:

$$k(s,s^{\prime })=\sigma_{p}^{2}x^{T}x^{\prime }$$ (4)

where *σ*_*p*_ is a hyperparameter that determines the prior variance of the fitness landscape. The squared exponential kernel is defined as:

$$k(s,s^{\prime })=\sigma_{p}^{2}exp\left(-\frac{{\left\|x-x^{\prime }\right\|}_{2}^{2}}{l}\right)$$ (5)

where *l* and *σ*_*p*_ are also hyperparameters and |∙|_2_ is the L2 norm. Finally, the Matérn kernel with $v=\frac{5}{2}$ is defined as:

$$k(s,s^{\prime })=\left(1+\frac{\sqrt{5{\left\|x-x^{\prime }\right\|}_{2}^{2}}}{l}+\frac{5{\left\|x-x^{\prime }\right\|}_{2}^{2}}{3l^{2}}\right)exp\left(-\frac{\sqrt{5{\left\|x-x^{\prime }\right\|}_{2}^{2}}}{l}\right)$$ (6)

Where *l* is once again a hyperparameter.

#### L1 regression feature identification and weighting

To identify those contacts in the ChR structure most important in determining chimera function (here, localization) we used L1 regression. Given the nature of our library design and the limited set of chimeras tested, there are certain residues and contacts that covary within our training set. The effects of these covarying residues and contacts cannot be isolated from one another using this data set and therefore must be weighted together for their overall contribution to ChR function. By using the concatenated sequence and structure binary feature vector for the training set we were able to identify residues and contacts that covary. Each individual set of covarying residues and contacts was combined into a single feature. L1 linear regression was then used to weight features as either zero or non-zero in their contribution to ChR function. The level of regularization was chosen by LOO cross-validation. We then performed Bayesian ridge linear regression on features with non-zero L1 regression weights using the default settings in scikit-learn [42]. The Bayesian ridge linear regression weights were plotted onto the C1C2 structure to highlight positive and negative contributions to ChR localization (**Fig 5**) and ChR expression (**S11 Fig**).

## Supporting information

S1 Data Localization and expression characterization of ChR chimeras predicted by the models. Measured localization and expression properties for each chimera tested and associated chimera_name, set, number of mutations, chimera_block_ID, and sequence. Chimera names and chimera_block_ID begin with either ‘c’ or ‘n’ to indicate the contiguous or non-contiguous library. The following 10 digits in the chimera_block_ID indicate, in block order, the parent that contributes each of the 10 blocks (‘0’:CheRiff, ‘1’:C1C2, and ‘2’:CsChrimR). For the contiguous library, blocks in the chimera_block_ID are listed from N- to C-termini; for the non-contiguous library the block order is arbitrary. The set for which the chimera was generated is listed. The number of mutations (m) from the closest parent for each chimera is included. Sequences list only the ChR open reading frame, the C-terminal trafficking and mKate2.5 sequences have been removed. The table shows mean properties (mKate_mean, GFP_mean, and intensity_ratio_mean) and the standard deviation of properties (mKate_std, GFP_std, and intensity_ratio_std). ND: not detected, below the limit of detection for our assay.
(CSV)

S1 Fig Chimera sequences in training set and their expression, localization, and localization efficiencies. (**A**) (top) shows blocks (different colors) for the contiguous (contig) and non-contiguous (non-contig) library designs and also shows block boundaries (white lines) for the combined contiguous and non-contiguous library designs on the three parental ChRs aligned with a schematic of the ChR secondary structure. (bottom) Sequences of training set chimeras showing block identities. The colors represent the parental origin of the block (red–CsChrimR, green–C1C2, and blue–CheRiff). (**B**) Cumulative distributions of the measured expression, localization, and localization efficiency of all 218 chimeras with the three parental constructs highlighted in color (5).
(TIF)

S2 Fig Chimera expression and localization cannot be predicted from simple rules. Expression and localization measurements are plotted with chimeras grouped based on (**A**) signal peptide sequence identity and (**B**) hydrophobicity in the transmembrane (TM) domains. (**A**) Each chimera in the training set is grouped based on its signal peptide identity, which could be the CheRiff (0), C1C2 (1), or CsChrimR (2) signal peptide. The measured expression and localization are shown for each chimera in each of the three groups. (**B**) The measured expression and localization with respect to the calculated level of hydrophobicity within the 7-TM domains of each chimera. Hydrophobicity was calculated in the region of the protein highlighted in the surface rendering on the ChR structure.
(TIF)

S3 Fig GP binary classification model for localization efficiency. Plots of predicted probability vs measured localization efficiency are divided into ‘high’ performers (white background) and ‘low’ performers (gray background) for localization efficiency. (**A**) Predicted probability vs measured localization efficiency for the training set (gray points) and the exploration set (cyan points). Predictions for the training and exploration sets were made using LOO cross-validation. (**B**) Predicted probabilities vs measured localization efficiency for the verification set. Predictions for the verification set were made by a model trained on the training and exploration sets. (**C**) Probability of ‘high’ localization efficiency for all chimeras in the recombination library (118,098 chimeras) made by a model trained on the data from the training and exploration sets. The gray line shows all chimeras in the library, the gray points indicate the training set, the cyan points indicate the exploration set, the purple points indicate the verification set, and the yellow points indicate the parents. For all plots, the measured localization efficiency is plotted on a log_2_ scale.
(TIF)

S4 Fig Chimera block identities for exploration, verification, and optimization sets. Block identity of chimeras from each set ranked according to their performance for localization with the best ranking chimera listed at the top of the list. ‘High’ and ‘low’ indicates those chimeras had a high predicted probability of localization vs a low predicted probability of localization. Each row represents a chimera. The three different colors represent blocks from the three different parents (red–CsChrimR, green–C1C2, and blue–CheRiff). The number of mutations from the nearest parent and the number of mutations from the nearest previously tested chimera from the library are shown for each chimera.
(TIF)

S5 Fig ROC curves for GP classification expression, localization, and localization efficiency models. ROC curves show true positive rate vs false positive rate for predictions from the expression (**A**), localization (**B**), and localization efficiency (**C**) classification models. The gray line shows the ROC for the combined training and exploration sets. The purple line shows the ROC for the verification set. The verification sets consist exclusively of chimeras with ‘high’ expression so no verification ROC curve for expression is shown. Predictions for the training and exploration sets were made using LOO cross-validation, while predictions for the verification set were made by a model trained on the training and exploration sets. Calculated AUC values are shown in the figure key.
(TIF)

S6 Fig Comparison of measured expression and localization efficiency for each data set. Swarm plots of expression (**A**) and localization efficiency (**B**) measurements for each data set compared with parents: training set, exploration set, verification set, and optimization set.
(TIF)

S7 Fig Cell population distributions of expression, localization, and localization efficiency properties for each chimera in the verification and optimization sets compared with parents. The distribution of expression (**A**), localization (**B**), and localization efficiency (**C**) for the population of transfected cells is plotted for each parent (top row), each chimera in the verification set (middle row), and each chimera in the optimization set (bottom row) using kernel density estimation for smoothing. Parents are plotted in red (CsChrimR), green (C1C2), and blue (CheRiff). Chimeras in the verification set are plotted in gray if they were predicted to be ‘low’ or purple if they were predicted to be ‘high’ in each property. The vertical, gray, dashed line indicates the mean behavior of the CheRiff parent for each property.
(TIF)

S8 Fig Predictive ability of GP localization models as a function of training set size. We trained GP models on random training sets of various sizes sampled from our data and evaluated their predictive performance on a fixed test set of sequences for the classification (**A**) and regression (**B**) localization models. The predictive performance of the classification model is described by AUC for the test set (**A**), while the predictive performance of the regression model (**B**) is described by the correlation coefficient (R-value) for the test set. For each training set size, the results are averaged over 100 random samples.
(TIF)

S9 Fig Important features for prediction of ChR localization aligned with chimeras with optimal localization. Features with positive weights from the localization model (**Fig 5**) are displayed on the C1C2 crystal structure which is colored based on the block design of two different chimeras, (**A**) n1_7 and (**B**) n4_7, from the optimization set. Features can be residues (spheres) or contacts (sticks) from one or more parent ChRs. Features/blocks from CsChrimR are shown in red, features/blocks from C1C2 are shown in green, and features/blocks from CheRiff are shown in blue. Gray positions are conserved residues. Sticks connect the beta carbons of contacting residues (or alpha carbon in the case of glycine). The size of the spheres and the thickness of the sticks are proportional to the parameter weights.
(TIF)

S10 Fig GP regression model for ChR expression. Shows the GP regression model predicted vs measured expression for the combined training and exploration sets (gray points). Predictions for the training and exploration sets were made using LOO cross-validation. The predicted and measured expression are plotted on a log_2_ scale. The combined training and exploration sets showed good correlation (R > 0.70).
(TIF)

S11 Fig Sequence and structure features important for prediction of ChR expression. Features with positive (**A**) and negative (**B**) weights are displayed on the C1C2 crystal structure (grey). Features can be residues (spheres) or contacts (sticks) from one or more parent ChRs. Features from CsChrimR are shown in red, features from C1C2 are shown in green, and features from CheRiff are shown in blue. In cases where a feature is present in two parents, the following color priorities were used for consistency: red above green above blue. Sticks connect the beta carbons of contacting residues (or alpha carbon in the case of glycine). The size of the spheres and the thickness of the sticks are proportional to the parameter weights. Two residues in contact can be from the same or different parents. Single-color contacts occur when both contributing residues are from the same parent. Multi-color contacts occur when residues from different parents are in contact. The N-terminal domain (NTD), C-terminal domain (CTD), and the seven transmembrane helices (TM1-7) are labeled.
(TIF)

S12 Fig Localization of engineered CbChR1 variant chimera 3c. Representative cell confocal images of mKate expression and GFP labeled localization of CsCbChR1 compared with top-performing CsCbChR1 single-block-swap chimera (chimera 3c), and top-performing parent (CsChrimR). CsCbChR1 shows weak expression and no localization, while chimera 3c expresses well and clearly localizes to the plasma membrane as does CsChrimR. Gain was adjusted in CsCbChR1 images to show any low signal. Scale bar: 10 μm.
(TIF)
