# Supplementary figures and images for: Machine learning to design integral membrane channelrhodopsins for efficient eukaryotic expression and plasma membrane localization
Source: PLoS Comput Biol. 2017 Oct 23;13(10):e1005786. doi: 10.1371/journal.pcbi.1005786 (PMC5695628; doi:10.1371/journal.pcbi.1005786)

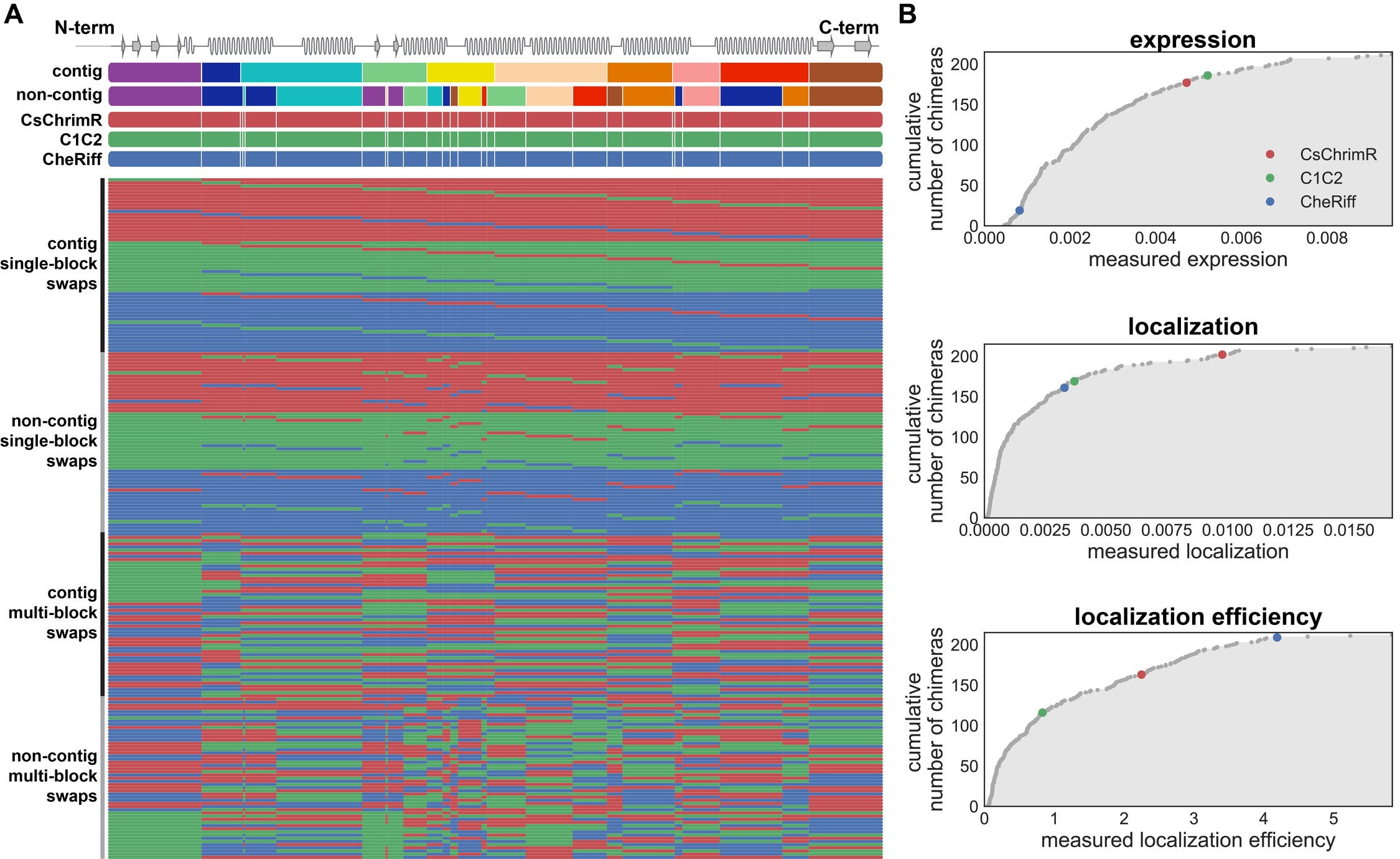

Supplement: S1 Fig — (A) (top) shows blocks (different colors) for the contiguous (contig) and non-contiguous (non-contig) library designs and also shows block boundaries (white lines) for the combined contiguous and non-contiguous library designs on the three parental ChRs aligned with a schematic of the ChR secondary structure. (bottom) Sequences of training set chimeras showing block identities. The colors represent the parental origin of the block (red–CsChrimR, green–C1C2, and blue–CheRiff). (B) Cumulative distributions of the measured expression, localization, and localization efficiency of all 218 chimeras with the three parental constructs highlighted in color (5). (TIF) [file pcbi.1005786.s002.tif]

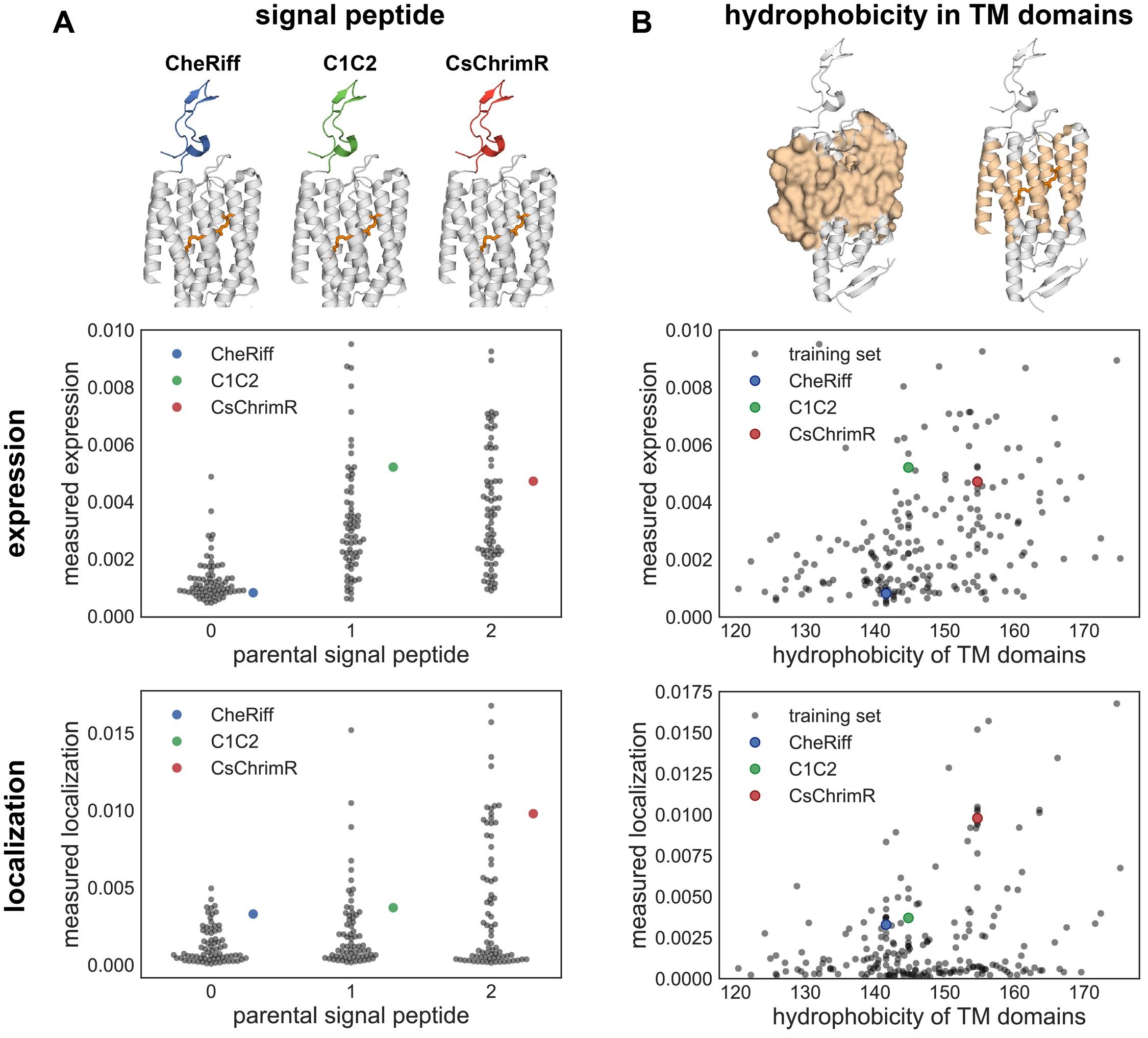

Supplement: S2 Fig — Expression and localization measurements are plotted with chimeras grouped based on (A) signal peptide sequence identity and (B) hydrophobicity in the transmembrane (TM) domains. (A) Each chimera in the training set is grouped based on its signal peptide identity, which could be the CheRiff (0), C1C2 (1), or CsChrimR (2) signal peptide. The measured expression and localization are shown for each chimera in each of the three groups. (B) The measured expression and localization with respect to the calculated level of hydrophobicity within the 7-TM domains of each chimera. Hydrophobicity was calculated in the region of the protein highlighted in the surface rendering on the ChR structure. (TIF) [file pcbi.1005786.s003.tif]

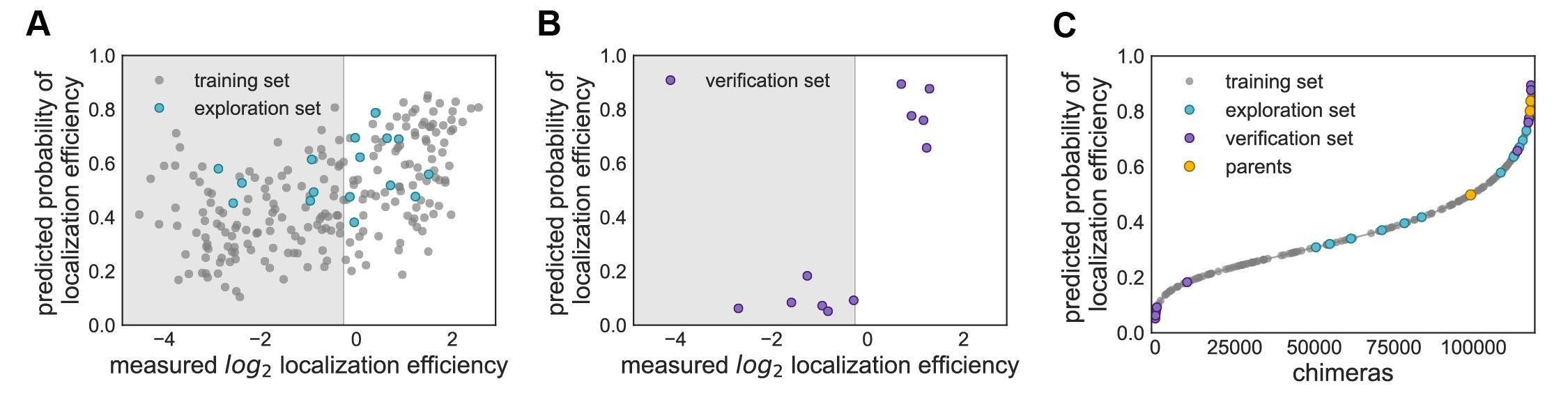

Supplement: S3 Fig — Plots of predicted probability vs measured localization efficiency are divided into ‘high’ performers (white background) and ‘low’ performers (gray background) for localization efficiency. (A) Predicted probability vs measured localization efficiency for the training set (gray points) and the exploration set (cyan points). Predictions for the training and exploration sets were made using LOO cross-validation. (B) Predicted probabilities vs measured localization efficiency for the verification set. Predictions for the verification set were made by a model trained on the training and exploration sets. (C) Probability of ‘high’ localization efficiency for all chimeras in the recombination library (118,098 chimeras) made by a model trained on the data from the training and exploration sets. The gray line shows all chimeras in the library, the gray points indicate the training set, the cyan points indicate the exploration set, the purple points indicate the verification set, and the yellow points indicate the parents. For all plots, the measured localization efficiency is plotted on a log2 scale. (TIF) [file pcbi.1005786.s004.tif]

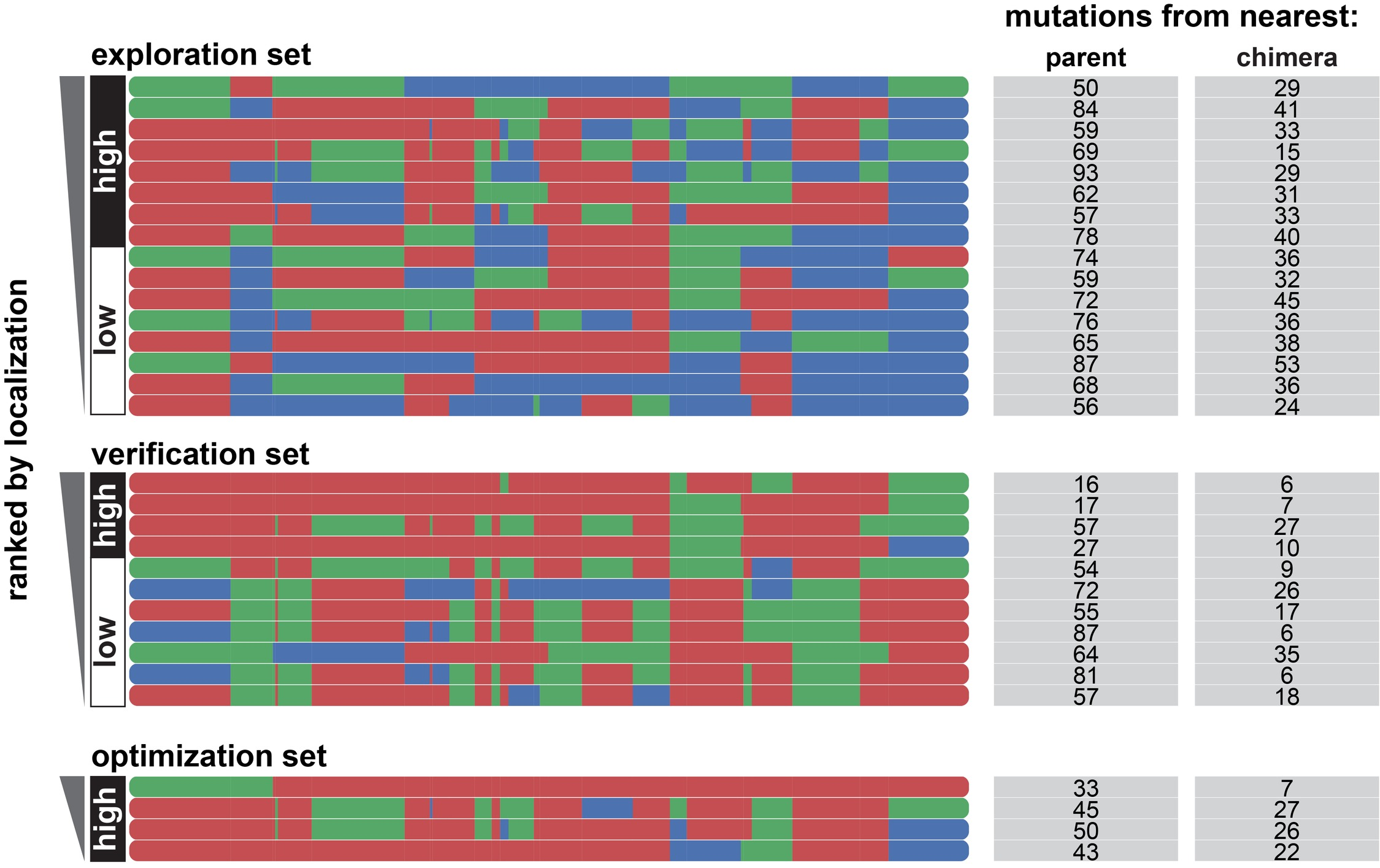

Supplement: S4 Fig — Block identity of chimeras from each set ranked according to their performance for localization with the best ranking chimera listed at the top of the list. ‘High’ and ‘low’ indicates those chimeras had a high predicted probability of localization vs a low predicted probability of localization. Each row represents a chimera. The three different colors represent blocks from the three different parents (red–CsChrimR, green–C1C2, and blue–CheRiff). The number of mutations from the nearest parent and the number of mutations from the nearest previously tested chimera from the library are shown for each chimera. (TIF) [file pcbi.1005786.s005.tif]

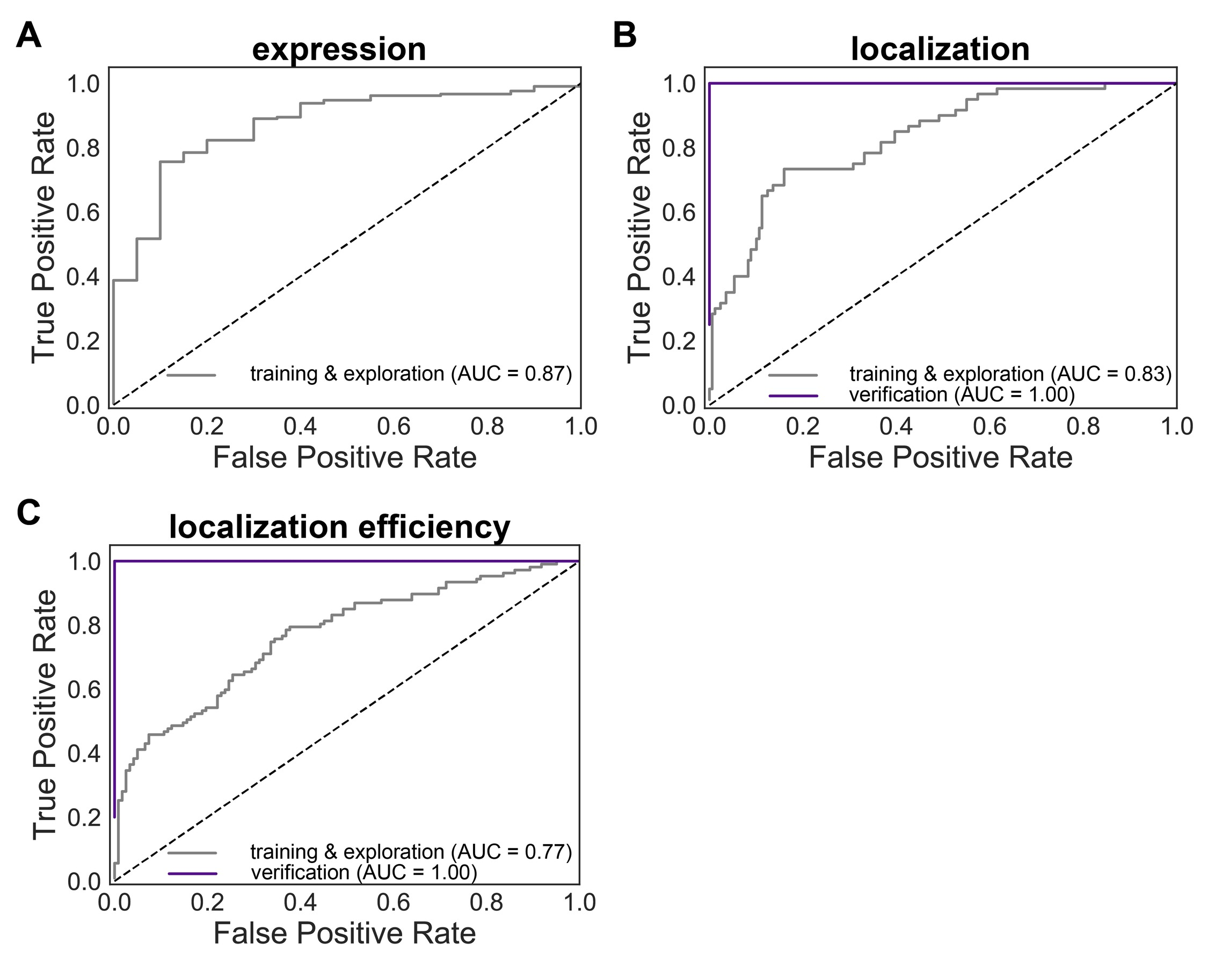

Supplement: S5 Fig — ROC curves show true positive rate vs false positive rate for predictions from the expression (A), localization (B), and localization efficiency (C) classification models. The gray line shows the ROC for the combined training and exploration sets. The purple line shows the ROC for the verification set. The verification sets consist exclusively of chimeras with ‘high’ expression so no verification ROC curve for expression is shown. Predictions for the training and exploration sets were made using LOO cross-validation, while predictions for the verification set were made by a model trained on the training and exploration sets. Calculated AUC values are shown in the figure key. (TIF) [file pcbi.1005786.s006.tif]

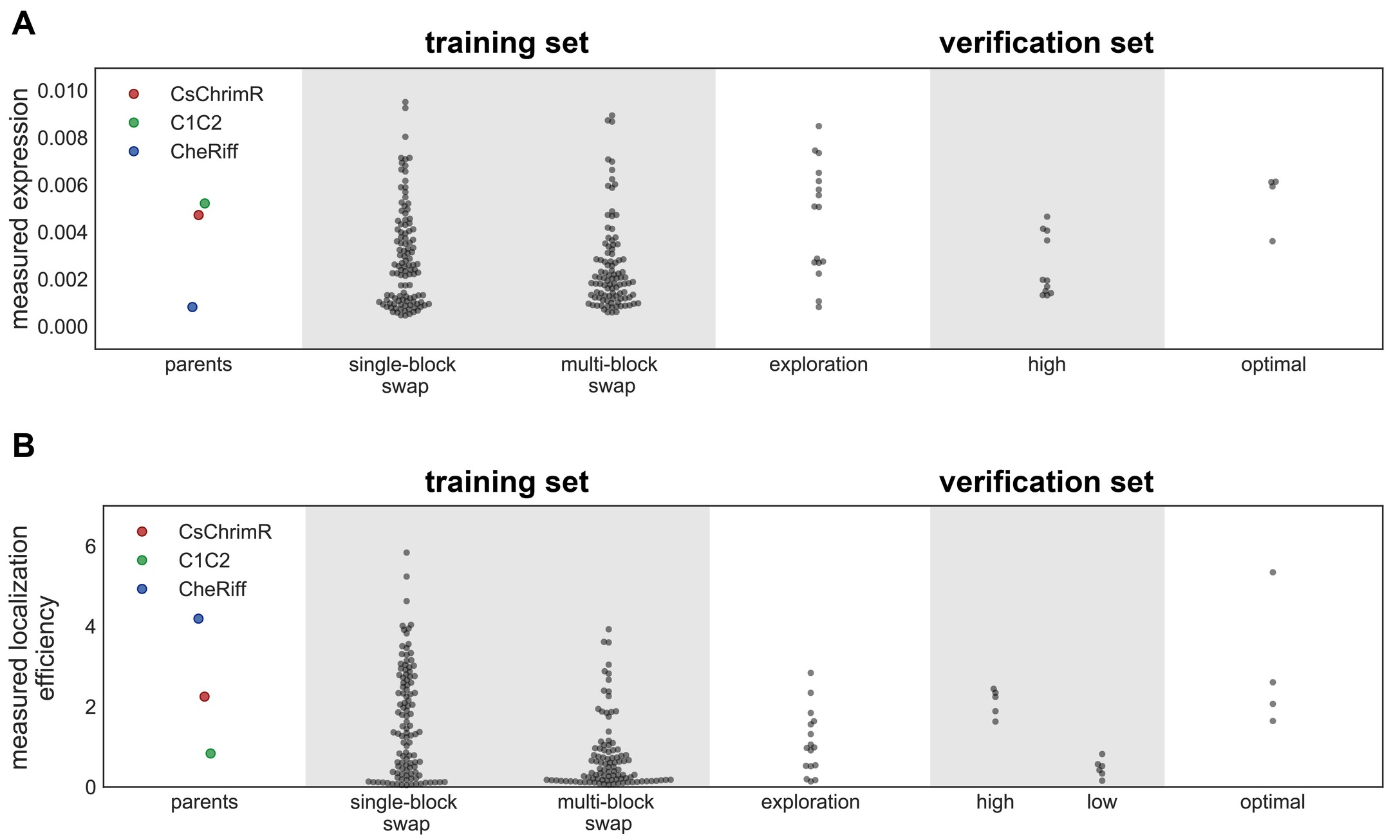

Supplement: S6 Fig — Swarm plots of expression (A) and localization efficiency (B) measurements for each data set compared with parents: training set, exploration set, verification set, and optimization set. (TIF) [file pcbi.1005786.s007.tif]

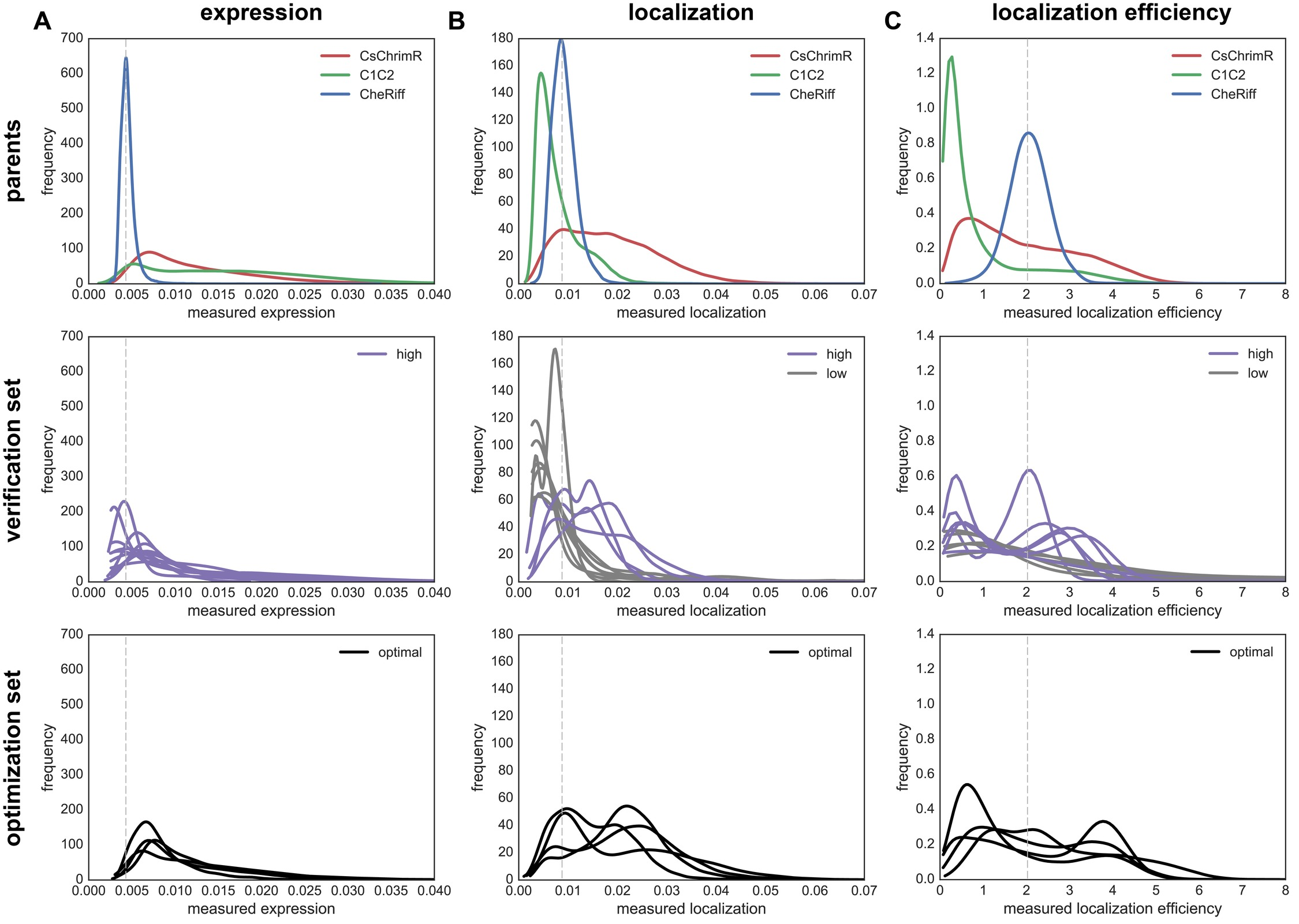

Supplement: S7 Fig — The distribution of expression (A), localization (B), and localization efficiency (C) for the population of transfected cells is plotted for each parent (top row), each chimera in the verification set (middle row), and each chimera in the optimization set (bottom row) using kernel density estimation for smoothing. Parents are plotted in red (CsChrimR), green (C1C2), and blue (CheRiff). Chimeras in the verification set are plotted in gray if they were predicted to be ‘low’ or purple if they were predicted to be ‘high’ in each property. The vertical, gray, dashed line indicates the mean behavior of the CheRiff parent for each property. (TIF) [file pcbi.1005786.s008.tif]

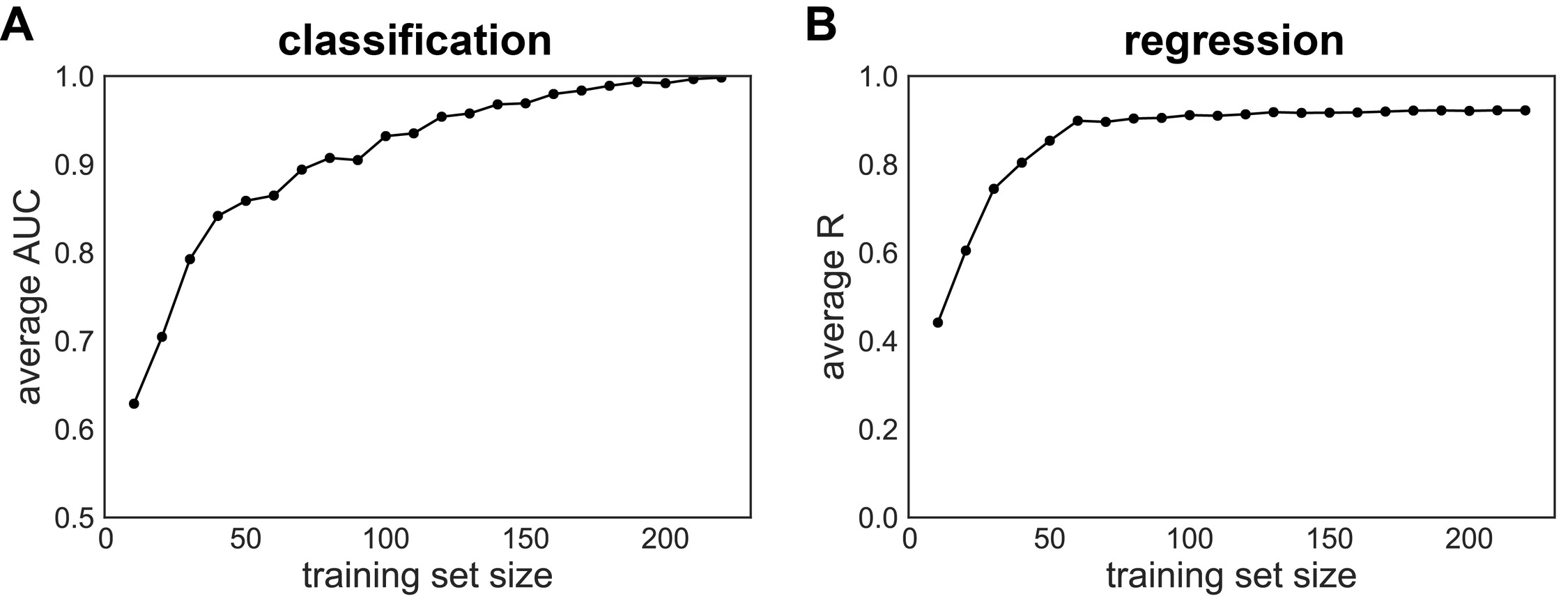

Supplement: S8 Fig — We trained GP models on random training sets of various sizes sampled from our data and evaluated their predictive performance on a fixed test set of sequences for the classification (A) and regression (B) localization models. The predictive performance of the classification model is described by AUC for the test set (A), while the predictive performance of the regression model (B) is described by the correlation coefficient (R-value) for the test set. For each training set size, the results are averaged over 100 random samples. (TIF) [file pcbi.1005786.s009.tif]

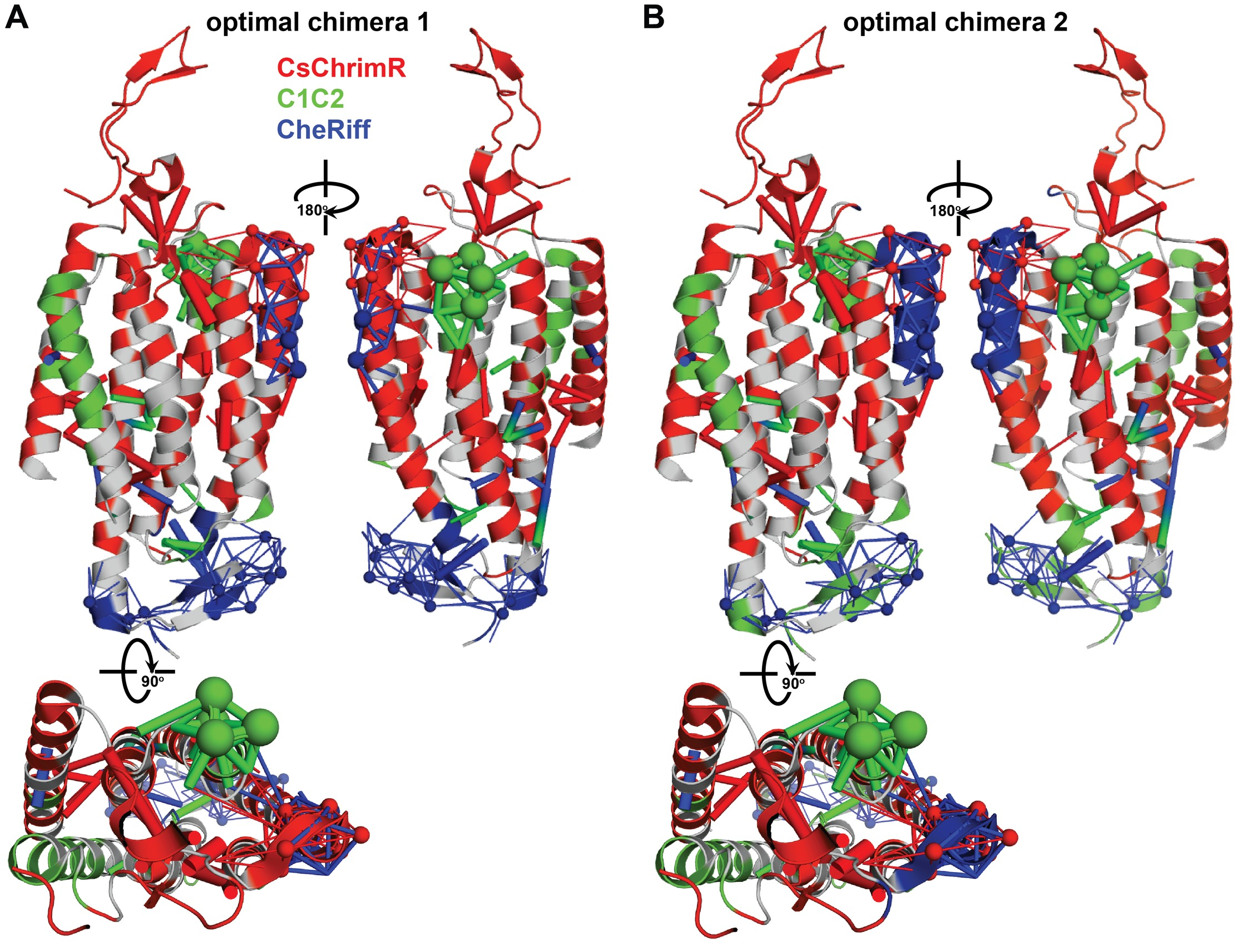

Supplement: S9 Fig — Features with positive weights from the localization model (Fig 5) are displayed on the C1C2 crystal structure which is colored based on the block design of two different chimeras, (A) n1_7 and (B) n4_7, from the optimization set. Features can be residues (spheres) or contacts (sticks) from one or more parent ChRs. Features/blocks from CsChrimR are shown in red, features/blocks from C1C2 are shown in green, and features/blocks from CheRiff are shown in blue. Gray positions are conserved residues. Sticks connect the beta carbons of contacting residues (or alpha carbon in the case of glycine). The size of the spheres and the thickness of the sticks are proportional to the parameter weights. (TIF) [file pcbi.1005786.s010.tif]

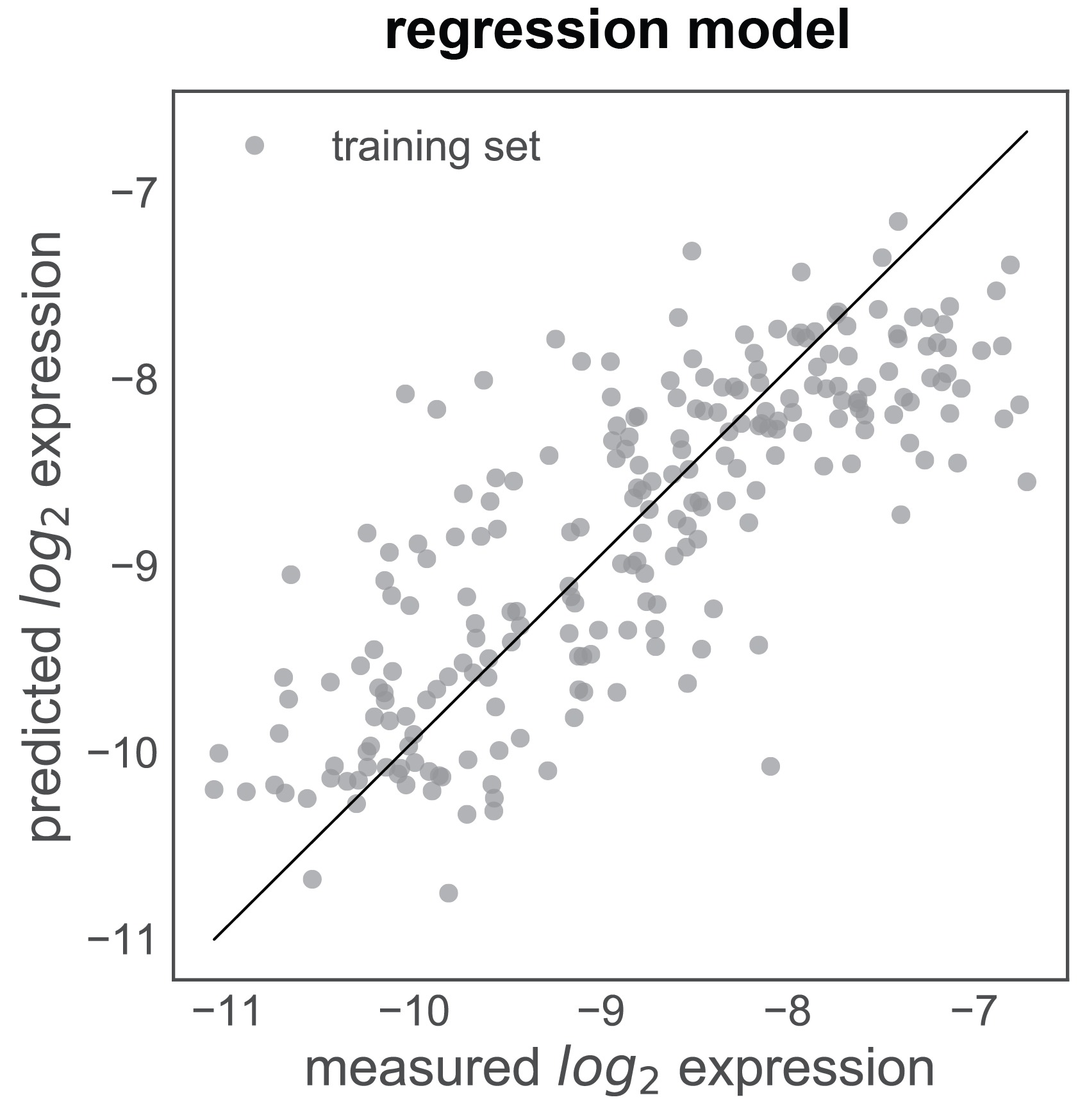

Supplement: S10 Fig — Shows the GP regression model predicted vs measured expression for the combined training and exploration sets (gray points). Predictions for the training and exploration sets were made using LOO cross-validation. The predicted and measured expression are plotted on a log2 scale. The combined training and exploration sets showed good correlation (R > 0.70). (TIF) [file pcbi.1005786.s011.tif]

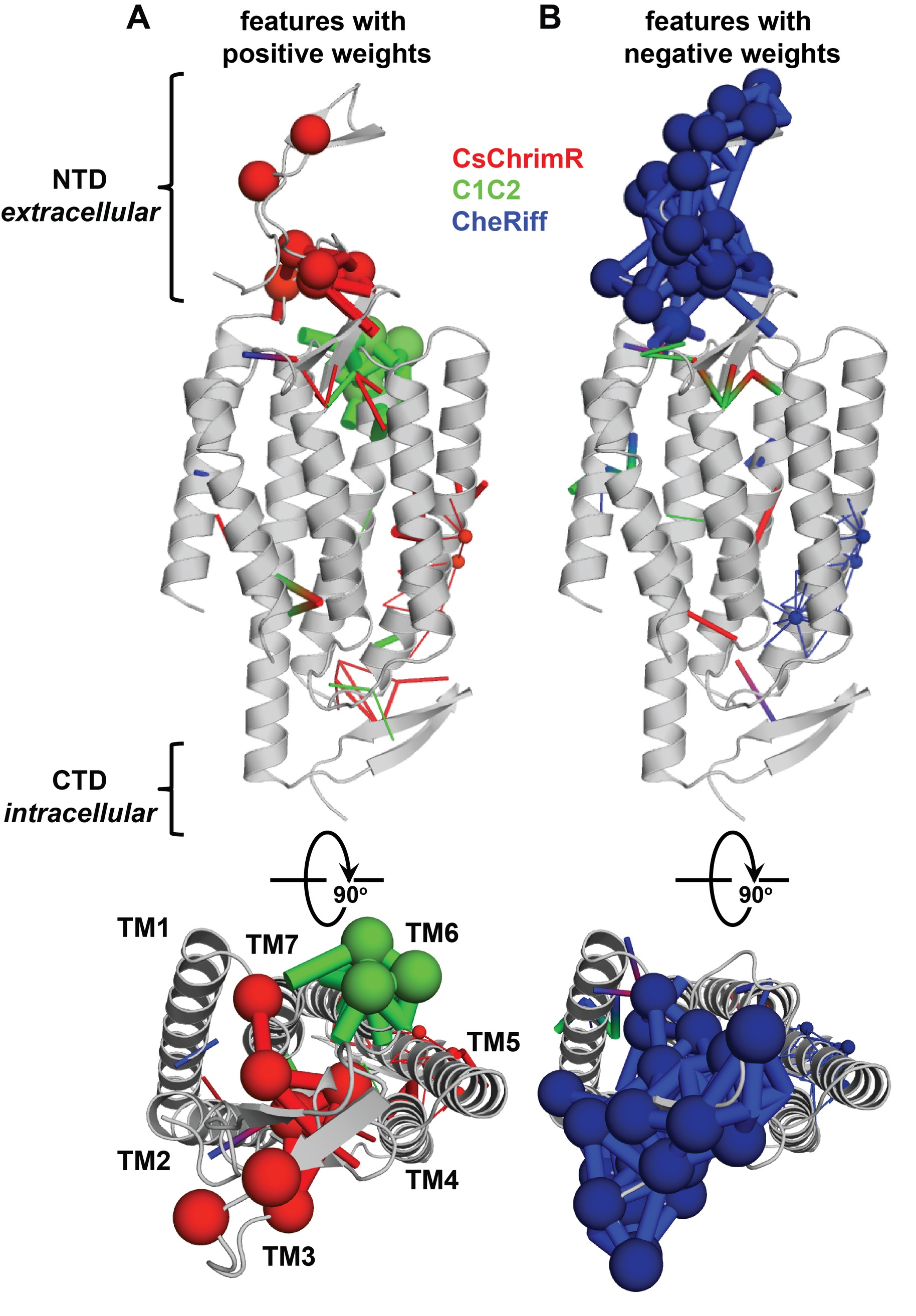

Supplement: S11 Fig — Features with positive (A) and negative (B) weights are displayed on the C1C2 crystal structure (grey). Features can be residues (spheres) or contacts (sticks) from one or more parent ChRs. Features from CsChrimR are shown in red, features from C1C2 are shown in green, and features from CheRiff are shown in blue. In cases where a feature is present in two parents, the following color priorities were used for consistency: red above green above blue. Sticks connect the beta carbons of contacting residues (or alpha carbon in the case of glycine). The size of the spheres and the thickness of the sticks are proportional to the parameter weights. Two residues in contact can be from the same or different parents. Single-color contacts occur when both contributing residues are from the same parent. Multi-color contacts occur when residues from different parents are in contact. The N-terminal domain (NTD), C-terminal domain (CTD), and the seven transmembrane helices (TM1-7) are labeled. (TIF) [file pcbi.1005786.s012.tif]

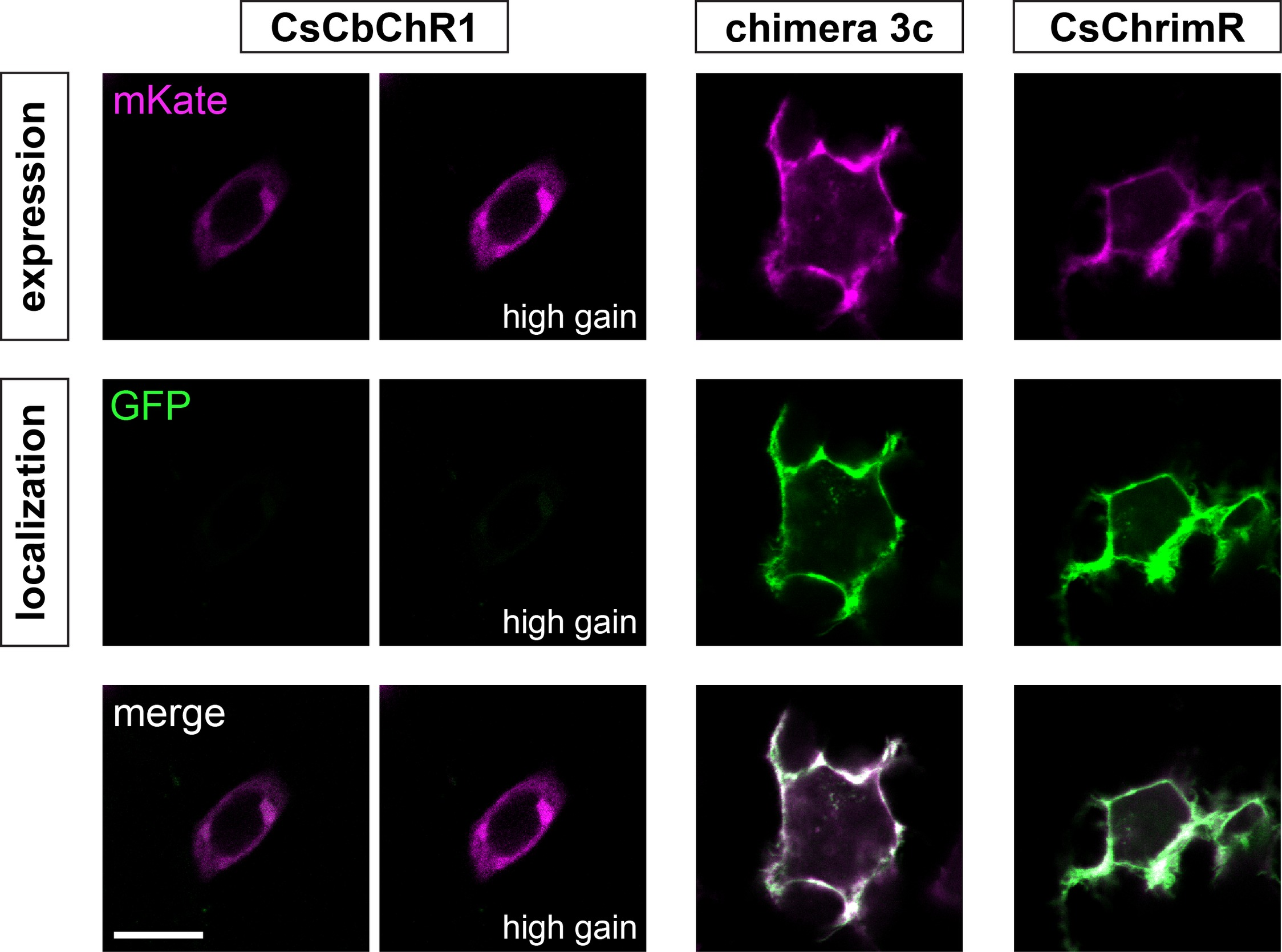

Supplement: S12 Fig — Representative cell confocal images of mKate expression and GFP labeled localization of CsCbChR1 compared with top-performing CsCbChR1 single-block-swap chimera (chimera 3c), and top-performing parent (CsChrimR). CsCbChR1 shows weak expression and no localization, while chimera 3c expresses well and clearly localizes to the plasma membrane as does CsChrimR. Gain was adjusted in CsCbChR1 images to show any low signal. Scale bar: 10 μm. (TIF) [file pcbi.1005786.s013.tif]
